# The transcription factor Traffic jam orchestrates the somatic piRNA pathway in *Drosophila* ovaries

**DOI:** 10.1016/j.celrep.2025.115453

**Published:** 2025-04-09

**Authors:** Azad Alizada, Aline Martins, Nolwenn Mouniée, Julia V. Rodriguez Suarez, Benjamin Bertin, Nathalie Gueguen, Vincent Mirouse, Anna-Maria Papameletiou, Austin J. Rivera, Nelson C. Lau, Abdou Akkouche, Stéphanie Maupetit-Méhouas, Gregory J. Hannon, Benjamin Czech Nicholson, Emilie Brasset

**Affiliations:** 1 iGReD, Université Clermont Auvergne, CNRS, INSERM, Faculté de Médecine, 63000 Clermont-Ferrand, France; 2 Cancer Research UK Cambridge Institute, University of Cambridge, Li Ka Shing Centre, Cambridge CB2 0RE, UK; 3 Department of Biochemistry and Cell Biology, Boston University Chobanian and Avedisian School of Medicine, Boston, MA, USA

## Abstract

The PIWI-interacting RNA (piRNA) pathway is essential for transposable element (TE) silencing in animal gonads. While the transcriptional regulation of piRNA pathway components in germ cells has been documented in mice and flies, their control in somatic cells of *Drosophila* ovaries remains unresolved. Here, we demonstrate that Traffic jam (Tj), the *Drosophila* ortholog of large Maf transcription factors in mammals, is a master regulator of the somatic piRNA pathway. Tj binds to regulatory regions of somatic piRNA factors and the major piRNA cluster *flamenco*, which carries a Tj-bound enhancer downstream of its promoter. Depletion of Tj in somatic follicle cells causes downregulation of piRNA factors, loss of *flamenco* expression, and derepression of *gypsy*-family TEs. We propose that the arms race between the host and TEs led to the co-evolution of promoters in piRNA pathway genes as well as TE regulatory regions, which both rely on a shared transcription factor.

## Graphical abstract

**Graphical abstract:**
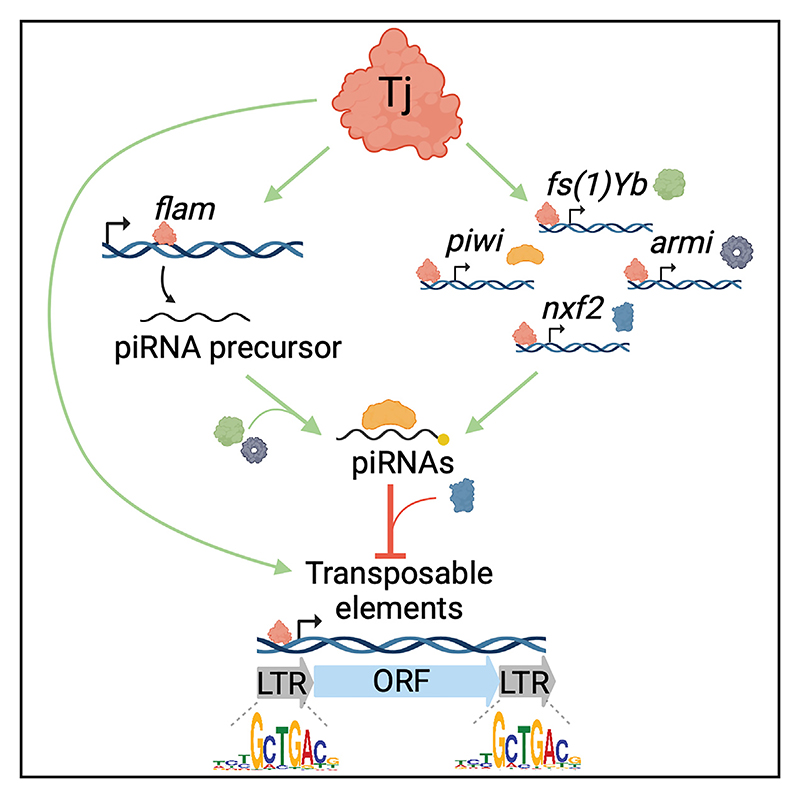

## Introduction

Transposable elements (TEs) are DNA sequences with the ability to move or replicate to new positions within the host genome.^1^ Succeeding waves of TE mobilization and repression have allowed TEs to accumulate in the genomes of nearly all organisms. Approximately 20% of the *Drosophila* genome and more than 50% of the human genome are composed of TE sequences.^2^ While their ability to transpose makes them powerful facilitators of genome evolution, their mobilization can also have deleterious effects, such as mutations, gene disruptions, and chromosomal rearrangements.^3,4^ The resulting selective pressure has driven the co-evolution of many mechanisms within the hosts to effectively counter the disruptive TE activity.^5^

In animals, the PIWI-interacting RNA (piRNA) pathway is one of the key defense mechanisms against active TEs. In *Drosophila*, the piRNA pathway is crucial for maintaining genome stability, particularly in gonadal tissues, which consist of germ cells surrounded by somatic follicle cells.^6–8^ Previous work has uncovered the existence of distinct piRNA pathways in germ cells and somatic follicle cells.^7–9^ Germ cells express a more sophisticated piRNA system that involves both post-transcriptional-silencing of transposon transcripts via the ping-pong loop and co-transcriptional silencing of nascent TEs. The first mechanism requires Aubergine (Aub), Argonaute-3 (Ago3), and numerous Tudor and RNA-binding proteins such as Qin, Tejas (Tej), Vasa (Vas), Krimper (Krimp), Spindle-E (Spn-E), and Brother of Yb (BoYb), whereas co-transcriptional TE silencing relies on P-element induced wimpy testis (Piwi), its cofactor Asterix (Arx), and the Panoramix (Panx)-induced co-transcriptional silencing (PICTS) complex composed of Panx, Nuclear RNA export factor 2 (Nxf2), NTF2-related export protein 1 (Nxt1), and Cut up (Ctp).^10–16^ Furthermore, piRNAs in germ cells are predominantly generated from precursor transcripts derived from discrete genomic loci called dual-strand piRNA clusters.^6,17–19^ The target repertoires of these piRNA clusters are dictated by both full-length and truncated transposon copies, and their transcription and export rely on non-canonical mechanisms involving Rhino (Rhi), Deadlock (Del), Cutoff (Cuff), Moonshiner (Moon), Nuclear export factor 3 (Nxf3), and Bootlegger (Boot).^6,17,20^

To avoid the robust silencing machinery in germ cells, some TEs, such as *gypsy* or *ZAM*, have gained the ability to express exclusively in the somatic follicle cells, form virus-like particles, and invade germ cells such as the oocyte.^21–23^ This infectious capacity enables their transposition into the germline genome and, hence, the transmission of new TE copies to future generations. As a consequence of this host-transposon arms race, somatic follicle cells have adapted a simplified piRNA pathway version comprised of the factors required for piRNA biogenesis (e.g., Armitage [Armi], Zucchini [Zuc], Minotaur [Mino], Gasz, Daedalus [Daed], Sister of Yb [SoYb], Vreteno [Vret], Shutdown [Shu], and the soma-specific Female sterile [1] Yb [Fs(1)Yb]) and co-transcriptional transposon silencing (e.g., Piwi, Arx, Panx, Nxf2, Nxt1, and Ctp).^8,13,14,24–28^ In addition, somatic piRNA clusters have emerged to control TE expression in the somatic follicle cells of the ovary. In contrast to the germline-active dual-strand piRNA clusters, the soma-expressed piRNA clusters are unistrand and give rise to piRNAs capable of targeting active TEs. The major somatic piRNA cluster in *Drosophila* ovaries is *flamenco* (*flam*).^6,29^ Unlike dual-strand piRNA clusters, *flam* is conserved across millions of years of drosophilid evolution^30^ and transcribed by a canonical mechanism that includes a promoter, splicing, and polyadenylation.^31^ Furthermore, to date, *flam* is the only known essential piRNA cluster, with *flam* mutants showing rudimentary ovaries and complete female sterility.^18,32^

Despite the importance of the somatic piRNA pathway, including the *flam* cluster and the somatic piRNA factors, the gene-regulatory circuit controlling their transcription remains to be determined. A recent study has shown that the transcription factor (TF) Ovo plays a key role in coordinating the expression of germline-specific piRNA pathway genes in the ovaries.^33^ However, it remains unclear whether one or multiple TFs drive the somatic piRNA pathway in the ovarian follicle cells. Here, we uncover *Traffic jam* (Tj), a large Maf TF, as a master regulator of the somatic piRNA pathway. Tj is critical for gonad development^34^ and was previously implicated in the regulation of *piwi* expression in somatic cells.^35^ In this study, we extend these findings by showing that Tj regulates the expression of many somatic piRNA pathway factors, as well as the key unistrand piRNA cluster *flam*. We demonstrate that Tj directly binds to the regulatory regions of these genes, driving their soma-specific expression in ovarian follicle cells. Furthermore, we identify a specific enhancer region downstream of the *flam* transcription start site (TSS) that is essential for its transcriptional activity in somatic cells and harbors Tj binding sites. Our findings shed light on the intricate transcriptional control necessary for TE silencing in the ovarian soma, offering new insights into the regulation of the soma-specific piRNA pathway.

## Results

### The TF Tj controls somatic piRNA factor expression

**Figure 1:**
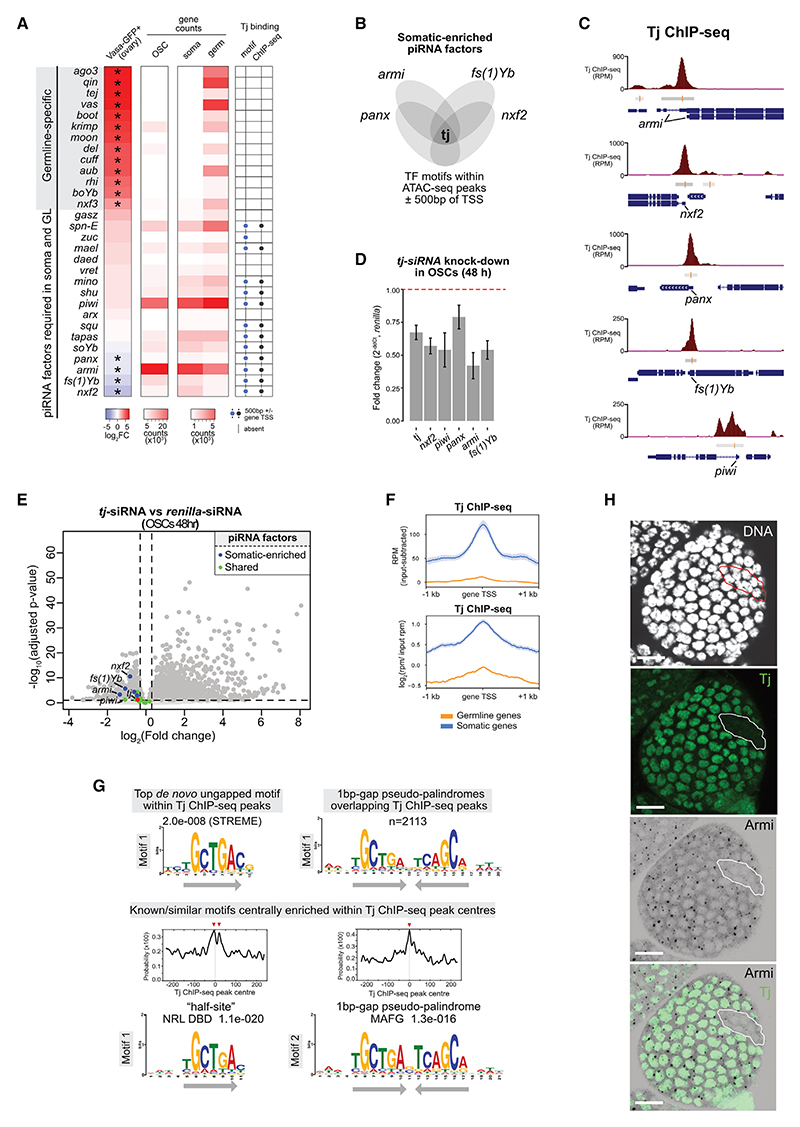
Traffic jam regulates the expression of somatic piRNA pathway genes in *Drosophila* ovarian somatic cells (A) Heatmap showing germline-specific and soma-expressed piRNA pathway genes ranked by gene expression fold enrichments in the fluorescence-activated cell-sorted germline cells (*vas*-GFP+) compared to somatic cells (*vas*-GFP−) dissociated from fly ovaries (*p < 0.01, DEseq2, RNA-seq *n* = 3 replicates from distinct samples). Normalized RNA-seq counts (DEseq2) from OSCs, vas-GFP+ germline cells, and vas-GFP−somatic cells are also shown. The presence of Tj motifs within ovary ATAC-seq peaks and Tj ChIP-seq peaks (ENCODE, female whole fly) within ±500 bp of a gene’s TSS is depicted with dots. (B) Venn diagram showing Tj as the only motif shared between OSC ATAC-seq peaks of the promoter regions (±500 bp of TSS) of somatic-enriched piRNA pathway genes. The full TF motif list is shown in Table S1. (C) Tj ChIP-seq from whole female flies showing Tj binding events to the promoters of somatic piRNA pathway genes (dm6). Gray bars indicate Tj ChIP-seq peaks, with the central red lines showing peak summits. (D) RT-qPCR plot showing 48 h siRNA knockdown of *tj* in OSCs resulting in downregulation of somatic piRNA pathway genes (normalized to *rpL32*; *n* = 3 replicates from distinct samples; error bars indicate standard deviation). (E) Volcano plot showing differential RNA-seq analysis (DEseq2) between *tj* and *renilla* siRNA knockdowns (48 h; *n* = 3 replicates from distinct samples). Blue dots show soma-enriched piRNA pathway genes (*fs(1)Yb, nxf2, panx, soYb*, and *armi*), green dots show general piRNA factors (e.g., *piwi*), and red dots show *tj*. (F) Genomic binding profiles of Tj at the promoters of germline- and soma-enriched genes using normalized ChIP-seq (ENCODE; whole fly; *n* = 3) signals with input subtraction (top) and log_2_ of chip rpm/input rpm (bottom). (G) The top-scoring *de novo* motif within Tj ChIP-seq peaks identified using STREME (MEME-ChIP) (top left). The numbers and motif logo (MEME) of genomic sites matching the 1 bp gapped pseudo-palindromic motif (MA0659.1; JASPAR 2024) within Tj ChIP-seq peaks identified using FIMO tool (*p* < 1 × 10^−4^) (top right). Enrichment of the known vertebrate motifs within Tj ChIP-seq peak centers using CentriMo (MEME-ChIP) (bottom). (H) Confocal images of *tj*^*eo2*^ clonal egg chamber showing Armi (black) and Tj (green) protein expression via immunostaining. The clone is circled in white. Nuclei are labeled with DAPI (white). Scale bars: 10 μm.

To uncover the regulatory mechanisms underlying the expression of somatic piRNA pathway factors, we first performed differential gene expression analysis between germline and somatic cells derived from *Drosophila* ovaries. We dissected and dissociated ovaries of transgenic *D. melanogaster* flies expressing GFP specifically in germ cells (using the *vasa* promoter) and sorted the cells using fluorescence-activated cell sorting (FACS) into germline (*vas*-GFP+) and somatic (*vas*-GFP−) populations.^33^ We then extracted RNAs and generated RNA sequencing (RNA-seq) libraries before carrying out a differential gene expression analysis between germline and somatic cells. This approach identified piRNA pathway genes with significant expression in the soma of the *Drosophila* ovary, namely *fs(1) Yb, nxf2, panx*, and *armi* (Figure 1A, left). Among these factors, *fs(1)Yb* was reported to be exclusively expressed in somatic cells,^36^ while the other factors are known to also function in germ cells.^37^ As expected, these four soma-enriched piRNA factors also showed expression in ovarian somatic cells (OSCs) (Figure 1A), a cell line derived from ovary somatic follicle cells.^35,38^

Next, to identify the potential TFs controlling the expression of these somatic-enriched piRNA factors (*fs(1)Yb, nxf2, panx*, and *armi*), we analyzed assay for transposase-accessible chromatin sequencing (ATAC-seq) datafrom wild-type OSCs^33^ and performed motif scanning using the FIMO tool (match *p* < 0.001; in the MEME Suite) to look for TF motifs (the FlyFactorSurvey database) that were shared between their promoter ATAC-seq peaks (±500 bp of the TSSs) (Figure 1B; Table S1). Surprisingly, this analysis identified only a single TF motif shared between the promoter ATAC-seq peaks of the four soma-enriched piRNA factors: Tj. We confirmed *in vivo* Tj binding to the promoters of *fs(1)Yb, nxf2, panx*, and *armi* (Figure 1C) by reanalyzing publicly available Tj-GFP chromatin immunoprecipitation (ChIP)-seq (referred to as Tj ChIP-seq) data from whole female flies.^39^ Moreover, Tj ChIP-seq peaks and Tj motifs were also present within promoters of other somatic piRNA factors that are expressed in both somatic and germline cells, such as *piwi*, but these were absent in promoters of the germline-specific piRNA factors (Figures 1A, right, and 1C), suggesting that Tj could be specifically controlling somatic piRNA pathway genes.

To validate our hypothesis that Tj regulates soma-expressed piRNA factors, we performed *tj* knockdowns in OSCs, followed by RT-qPCR. Tj depletion resulted in the downregulation of somatic piRNA factors (Figure 1D). Although *tj* levels were reduced by ~40% following 48 h of knockdown, we observed similar levels of downregulation for *nxf2, piwi, armi*, and *fs(1)yb* (RT-qPCR, *n* = 3 replicates from distinct samples, *p* < 0.01) (Figure 1D). To corroborate these results, we performed RNA-seq following 48 and 96 h of *tj* knockdown. These also showed significant downregulation of the soma-enriched piRNA factors *fs(1) Yb, nxf2, panx, soYb*, and *armi* (RNA-seq, *n* ≥ 3 replicates from distinct samples, adjusted p value [*p*.adj.] < 0.01) (Figures 1E and S1A). The piRNA factors shared between the somatic and germline cells that showed Tj binding at their promoters *in vivo* (i.e., *piwi, vret, mino*, and *shu*) were similarly down-regulated upon *tj* knockdowns in OSCs (Figures 1E and S1A). These results suggest that Tj is a soma-enriched TF responsible for the regulation of soma-expressed piRNA pathway genes in *Drosophila* OSCs, likely via direct promoter binding.

Genome-wide Tj ChIP-seq analysis revealed 7,956 Tj binding sites overall, which were enriched at the promoters of soma-expressed genes (including the somatic piRNA pathway genes) and depleted from promoters of the germline-enriched genes, including germline-specific piRNA pathway genes (Figures 1A and 1F). Specifically, Tj peaks overlapped with 714 TSSs (within ±500 bp of the TSS) of 419 soma-expressed genes (defined using RNA-seq log_2_ fold change [log_2_FC] < −1, *p*.adj. < 0.01, *n* = 3 from fluorescence-activated cell-sorted *vas*-GFP−cells), representing 62% of all soma-enriched gene TSSs (*n* = 1,142) (Table S1). Motif analysis revealed that the top-scoring *de novo* motif within Tj ChIP-seq peaks had a consensus sequence of TGCTGAC (STREME e value = 2.0 × 10^−8^). We noticed that the top-enriched motif within the peak centers had a consensus sequence of TGCTGA (CentriMo e value = 1.1 × 10^−20^) and was often found nearby its reverse complement, TCAGCA (as close as by 1 bp spacing), resulting in a pseudo-palindromic TGCTGA(N)_n_TCAGCA motif (CentriMo e value = 1.3 × 10^−16^; FIMO *n* = 2,113 sites) (Figures 1G and S1B). Interestingly, this closely mirrors the Maf recognition element (MARE) motifs recognized by DNA-binding domains of the dimeric basic leucine zipper Maf family TFs,^40,41^ suggesting that Tj could bind some target sites as a dimer. Overall, our results indicate that Tj specifically directs transcriptional regulation of the somatic ovarian program, including somatic piRNA pathway components, by binding to TF motifs at the promoters of somatic target genes.

To test Tj’s role in regulating somatic piRNA factors in an *in vivo* setting, we disrupted Tj activity and examined the expression of piRNA factors through immunostaining in *Drosophila* adult ovaries (Figure 1H). As Tj plays a critical role in regulating the formation and function of the germline stem cell niche and its mutation leads to ovarian atrophy,^42,43^ we abolished Tj activity in mosaic clones of follicle cells carrying a genetic null allele of Tj, the *tj*^*eo2*^ allele.^34^ This allele carries a point mutation resulting in a premature stop codon, resulting in a truncated version of the Tj protein, which lacks critical DNA-binding domains, including the leucine zipper and nuclear localization signal.^34^ As expected, in wild-type follicle cells, Armi, a protein essential for piRNA biogenesis, accumulates in distinct foci representing Yb bodies near the nuclear periphery.^44^ However, in *tj* mutant clones, Armi proteins were completely absent (Figure 1H). This finding confirms that, as observed in OSCs, Tj regulates piRNA factor expression within follicle cells of the adult ovary.

### Tj binds to conserved motifs in the regulatory region of the piRNA cluster *flam*

**Figure 2:**
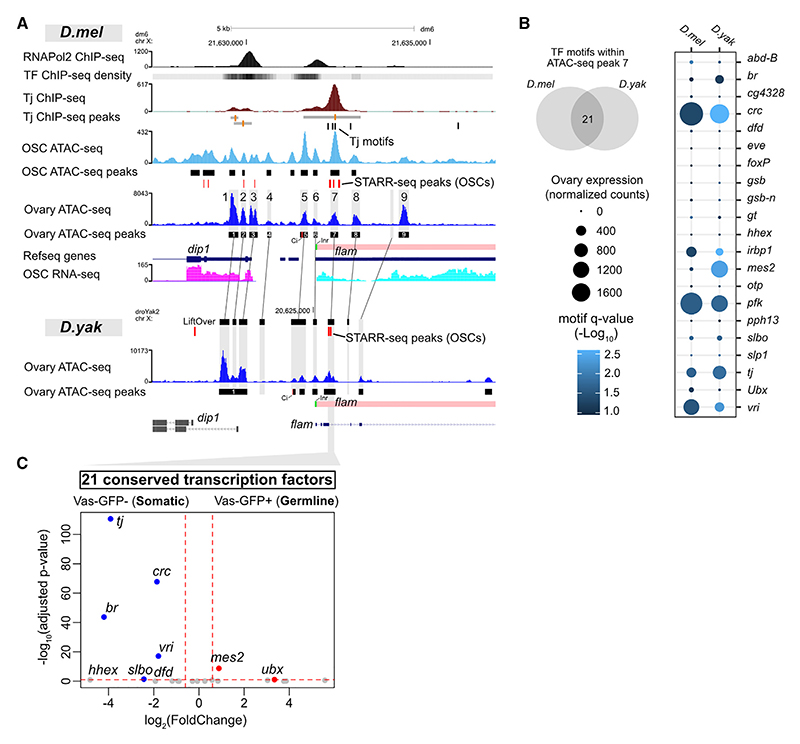
Tj binds to conserved *cis*-regulatory elements in *flamenco* (A) Conservation of ovary ATAC-seq peaks in the *flam* promoter region of *D. melanogaster* (*dm6*) and *D. yakuba* (*droYak2*) species (orthologous regions derived with UCSC LiftOver and chain files). OSC ATAC-seq peaks, OSC STARR-seq peaks, OSC RNA-seq, RNA polymerase II (RNA Pol II) ChIP-seq (ovary),^20^ and Tj ChIP-seq (ENCODE, whole fly) are shown. (B) Venn diagram showing motifs for 21 TFs shared between ATAC-seq peak #7 in *D. melanogaster* and its orthologous peak region in *D. yakuba*. Dot plot shows the expression of 21 candidate TFs in the ovaries of *D. melanogaster* and *D. yakuba* (read depth- and gene length-normalized DEseq2 counts, RNA-seq *n* = 4). (C) Soma and germline enrichment of 21 candidate TFs based on differential gene expression between somatic (*vas*-GFP−) and germline (*vas*-GFP+) cells from the fluorescence-activated cell-sorted fly ovaries (DEseq2 was performed between *vas*-GFP−[*n* = 3] and *vas*-GFP+ [*n* = 3] RNA-seq libraries using counts from all genes).

We next asked whether Tj also regulates somatic piRNA cluster transcription. The major piRNA cluster active in OSCs and somatic follicle cells is *flam*.^6,29^ Analysis of ovary and OSC ATAC-seq data^33,45^ revealed peaks upstream of *flam* that correspond to the nearby gene *DIP1* (ATAC-seq peaks #1–4), as well as three conserved peaks 1 kb upstream and 5 kb downstream of the *flam* TSS in *D. melanogaster* (ATAC-seq peaks #5–7) that were also present in the orthologous region in the *D. yakuba* genome (Figure 2A). Peak #5 harbored the motif of Cubitus interruptus (Ci), a TF previously reported to contribute to *flam* expression,^46^ while peak #6 contained the initiator element (Inr) of the *flam* TSS.^46^ Peak #8 overlapped with an alternative, further-downstream TSS that was previously identified by precision run-on sequencing (PRO-seq) (the second *flam* PRO-seq transcription initiation peak).^47^ We noted that the conserved peaks #5 and #7 had disproportionately stronger ATAC-seq signals in OSCs compared to the whole ovaries, suggesting their somatic enrichment (Figure 2A). To identify enhancer activity genome wide, we reanalyzed self-transcribing active regulatory region sequencing (STARR-seq) data performed in OSCs using genomic DNA of both *D. melanogaster* and *D. yakuba*.^48–50^ We found that the enhancer activity signal overlapped with ATAC-seq peak #7 in *D. melanogaster* and its corresponding orthologous peak in *D. yakuba* (Figure 2A). Motif scanning (FIMO tool) revealed 21 TF motifs (the FlyFactorSurvey database) that were shared between peak #7 in *D. melanogaster* and its orthologous region in *D. yakuba* (Figure 2B). Intriguingly, the motif for Tj was among the shared hits. RNA-seq data from the ovaries of *D. melanogaster* and *D. yakuba* were then used to reveal which TF candidates were expressed in the ovaries of both species (Figure 2B). Of these, four (*tj, crc, br*, and *vri*) showed enrichment in the ovarian soma, with *tj* being the top hit (Figure 2C).

In the *D. melanogaster* genome, Tj motifs were found downstream of the *flam* TSS at positions +327 to +332 (chrX:21,632,218–21,632,223; dm6), +437 to +450 (mutated pseudo-palindrome; chrX:21,632,328–21,632,340; dm6), +517 to +523 (summit of Tj ChIP-seq peak; chrX:21,632,408–21,632,414; dm6), and +935 to +940 (upstream of the alternative *flam* TSS; chrX: 21,632,826–21,632,831; dm6) bp (Figure 2A). Through Tj ChIP-seq analysis, we confirmed a strong Tj binding peak (−336 to +1,214 bp) overlapping these motifs. Interestingly, two of the Tj motifs (+437 to +450 and +517 to +523 bp) also overlapped with conserved ovary ATAC-seq peak #7 (Figure 2A). The summits of the Tj ChIP-seq peak, the ovary ATAC-seq peak, and the OSC ATAC-seq peak coincided with the +517 to +523 bp Tj motif (Figure 2A). Taken together, our results suggest that, in addition to regulating somatic piRNA genes, Tj may also control the piRNA cluster *flam*.

### Expression of *flam* in follicle cells is dependent on a regulatory region located downstream of its TSS

**Figure 3:**
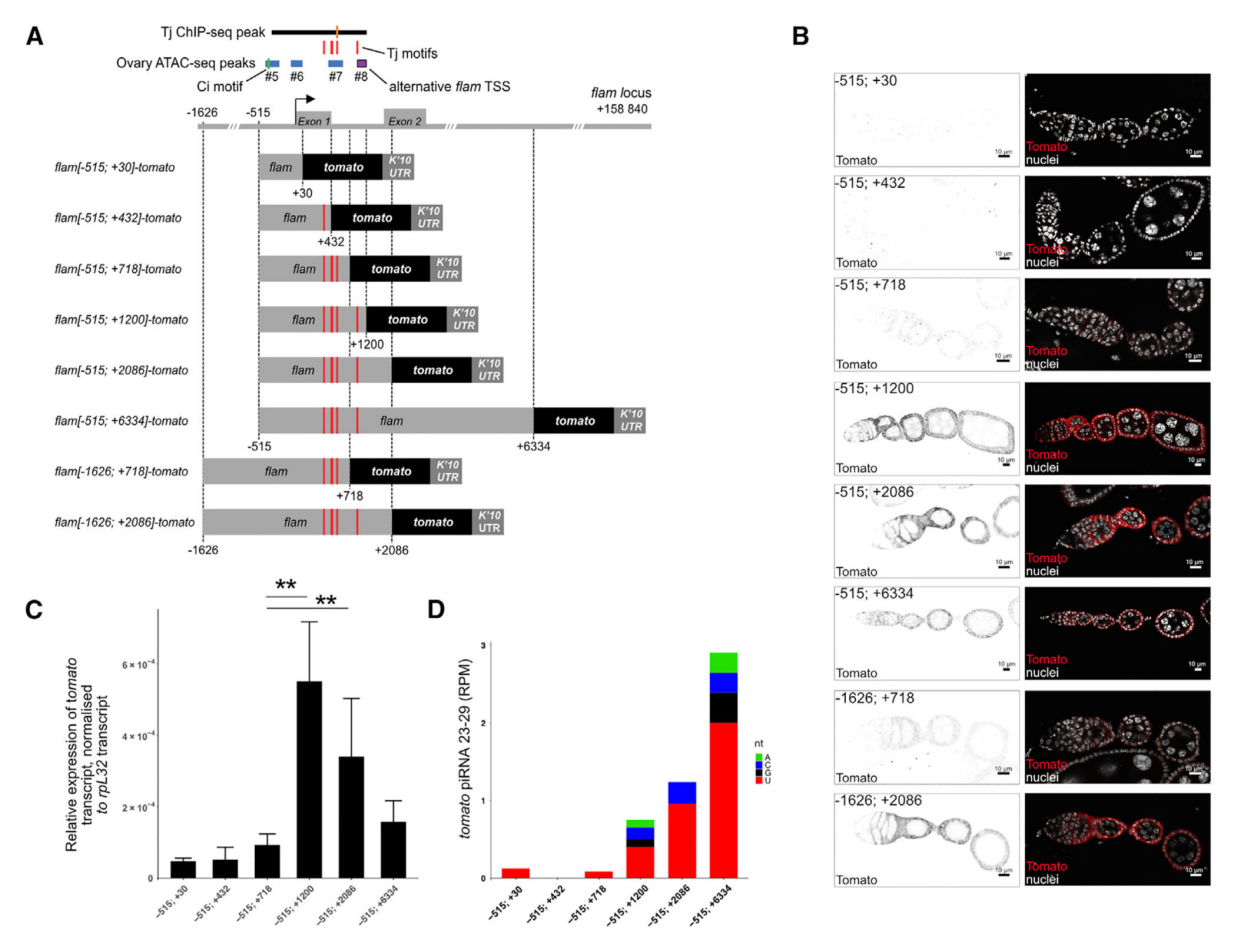
An enhancer of *flam* is located downstream of the TSS (A) Schematic representation of the transgenic *tomato* reporter constructs. The positions of the Tj ChIP-seq peak (line in the middle showing the peak summit), Tj motifs (red), and ATAC-seq peaks (#5–8) are highlighted on top. (B) Confocal images of ovarioles of the indicated reporters showing (left) Tomato immunostaining (gray) and (right) the merge of Tomato immunostaining (red) and nuclei stained with DAPI (white). Scale bars: 10 μm. (C) Relative transgene expression levels in ovaries measured by RT-qPCR. *tomato* expression was normalized to *rpL32*. Mean expression is shown (*n* = 6), with error bars representing standard deviation. Statistical significance was determined using the Wilcoxon-Mann-Whitney non-parametric test (**p < 0.01). (D) Amount of piRNAs produced from the *tomato* reporter sequence expressed in reads per million (RPM). Nucleotide biases of the first nucleotide of piRNAs are shown as indicated.

Previous work identified the major TSS and the minimal promoter of *flam* in ovarian somatic sheath (OSS) cells.^46^ To Cell Reports *44*, 115453, April 22, 2025 5 confirm the *flam* regulatory regions, including putative Tj motifs, *in vivo*, we generated transgenic flies carrying a transgene expressing the *tomato* reporter gene under the control of different *flam* promoter regions (starting at −1,626 bp upstream and ending at +6,334 bp downstream of the *flam* TSS) (Figure 3A). To compare the relative expression of these reporter constructs, we integrated all reporter variants into the identical genomic site at cytological position 53B2 and followed Tomato expression in fly ovaries by immunostaining and RT-qPCR (Figures 3A–3C). The strongest Tomato expression in somatic follicle cells was observed for the −515; +1,200 and −515; +2,086 constructs, with weaker signals found for the −515; +718 construct (Figures 3B and 3C). The increased Tomato expression observed in the −515; +1,200 line prompted us to further explore this region. The presence of the fourth Tj motif identified at +935 to +940 bp (TCAGCA), together with the alternative *flam* TSS immediately downstream of this region at +977 to +1,202 (ovary ATAC-seq peak #8 matching the second *flam* PRO-seq transcription initiation peak), potentially contributed to the enhanced Tomato expression observed when the region in constructs extends beyond +718.

Surprisingly, almost no artificial piRNAs matching tomato were detected with the −515; +432 and −515; +718 constructs (Figure 3D). These results suggest that the first 718 bp of the *flam* transcript, which were previously described as the piRNA trigger sequence in OSS cells,^51^ are not sufficient to direct artificial piRNA processing from the *tomato* reporter *in vivo* (Figure 3D). In contrast, a longer −515; +6,334 construct showed weaker Tomato expression but exhibited markedly stronger piRNA production relative to the −515; +1,200 and −515; +2,086 reporters 6 Cell Reports *44*, 115453, April 22, 2025 (Figures 3B–3D). Hence, including more *flam* sequences appeared to enhance piRNA processing efficiency and resulted in a concordant decrease of reporter messenger RNAs (mRNAs). This observation could be similar to the size-dependent dynamics observed in pachytene piRNA clusters in mice.^52^ As expected, no Tomato expression was detected in the germline regardless of the constructs used (Figure 3B).

**Figure 4:**
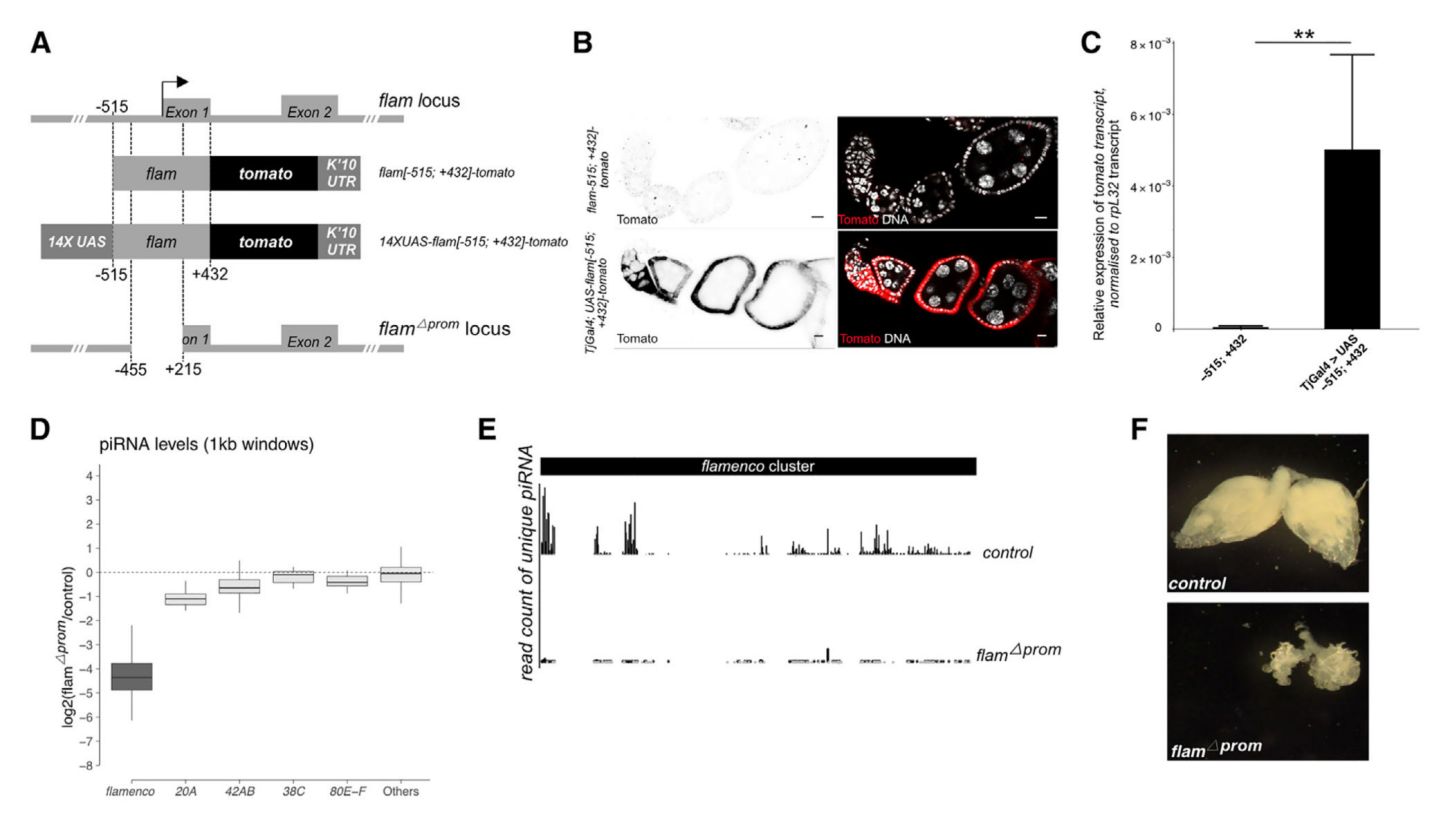
The minimal *flam* promoter is crucial for its expression and piRNA biogenesis (A) Schematic representation of the reporter constructs and *flam* promoter deletion (*flamΔprom* locus). (B) Confocal images of ovarioles of the indicated reporters showing Tomato (black, right; red, left) expression by immunostaining. Nuclei are labeled with DAPI (white). Scale bars: 10 μm. (C) Relative transgene expression levels in ovaries measured by RT-qPCR. *tomato* expression was normalized to *rpL32*. Mean expression is shown (*n* = 6), with error bars representing standard deviation. Statistical analysis was performed using the Wilcoxon-Mann-Whitney non-parametric test (**p < 0.01). (D) Boxplot showing changes in piRNA levels for the indicated clusters (split into 1 kb tiles) in *flam* mutants (*flamΔprom homozygote*) relative to control (*flamΔprom heterozygote*). The boxplot displays the median (line) and the first and third quartiles (box). (E) Genome uniquely mapping piRNAs (normalized per million of genome-mapped reads with 0 mismatches) are shown from control (*flamΔprom heterozygote*) (top) and *flam* mutant (*flamΔprom homozygote*) (bottom) ovaries. (F) Morphology of *Drosophila* ovaries from control (*flamΔprom heterozygote*) (top) and *flam* mutant (*flamΔprom homozygote*) (bottom) flies.

Neither the transgene carrying the −515; +30 *flam* regulatory region (which includes the minimal promoter) nor the reporter comprising −515; +432 (which includes the first exon) was sufficient to drive strong Tomato expression in somatic follicle cells (Figures 3B and 3C). These results suggest that while the Ci binding site (−394 to −366 bp from the *flam* TSS) is required for minimal *flam* expression in OSS cells, it is unable to induce strong expression in adult follicle cells. To confirm that the minimal *flam* promoter we have previously identified can efficiently drive transcription *in vivo*, we added UAS sequences in front of the −515; +432 *flam* promoter (Figure 4A). When combined with a follicle cell-specific *tj*-Gal4 driver, we indeed observed strong Tomato expression in somatic follicle cells (Figures 4B and 4C). Next, using CRISPR-Cas9, we deleted the minimal promoter region and tested its requirement for *flam* expression (Figures 4A and 4D–4F). The resulting *flam* mutants, named *flamΔprom*, were female sterile and exhibited fully atrophied ovaries, along with a loss of *flam* piRNAs, while the expression of the germline piRNA clusters remained unaffected (Figures 4D–4F). These results suggest that the −515; +432 region (relative to the *flam* TSS) contains the minimal promoter necessary for *flam* expression but not its full regulatory region. Furthermore, no Tomato expression was detected in the ovaries of transgenic flies carrying the +770; +2,086 *flam* region (Figures S2A and S2B). Overall, our findings suggest that the sequence between +432 and +1,200 bp downstream of the *flam* TSS that overlaps Tj ChIP-seq peaks and contains Tj motifs functions as an enhancer driving efficient soma-specific *flam* expression in the adult ovary.

We also investigated whether the sequence upstream of the *flam* TSS influences its expression by testing a larger *flam* promoter that includes the -1,624 bp region that ranges from the *flam* TSS to the closest upstream gene, *DIP1* (Figure 3A). This extended promoter did not enhance Tomato expression in the somatic follicle cells of the −1,624; +718 and −1,624; +2,086 transgenic lines (Figures 3A–3C). These findings suggest the absence of additional enhancers upstream of the minimal *flam* promoter.

### Tj is the master TF for follicle cell-specific expression of *flam*

**Figure 5:**
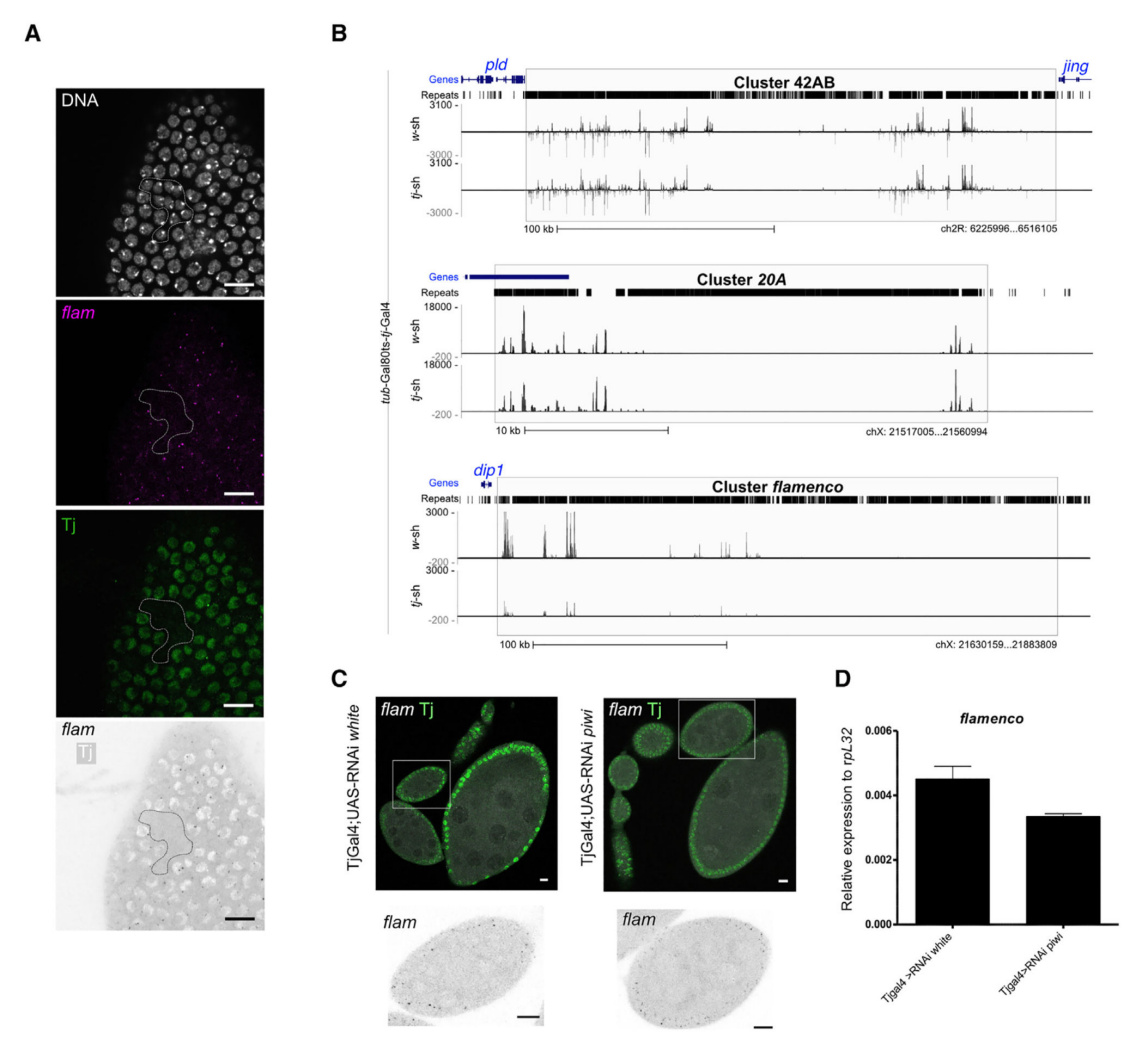
Tj controls *flam* expression (A) Confocal images of *tj*^*eo2*^ clonal egg chamber showing *flam* RNA (magenta) by smFISH RNA and Tj (green) by immunostaining. The clones are circled in white. Nuclei are labeled with DAPI (white). Scale bars: 10 μm. (B) Uniquely mapped piRNAs are plotted over the *42AB, 20A*, and *flam* piRNA clusters. The piRNA profiles of *tub*-Gal80ts-*tj*-Gal4; *w-sh* (control) and *tub*-Gal80ts-*tj*-Gal4; *tj-sh* adult ovaries are shown, normalized per million of unique genome-mapping piRNAs (0 mismatches). (C) Confocal images of egg chambers with indicated genotypes showing *flam* RNA (magenta) and Tj (green) by immunofluorescence FISH (immuno-FISH). A zoom on one egg chamber (white square) is shown on the bottom. Scale bars: 10 μm. (D) Relative transgene expression in the indicated ovaries as measured by RT-qPCR. *tomato* expression levels were normalized to the expression of *rpL32*. Mean expression is shown (*n* = 6), with error bars representing standard deviation.

To test for direct regulation of the *flam* piRNA cluster by Tj *in vivo*, we abolished Tj activity and monitored *flam* expression by single-molecule fluorescence *in situ* hybridization (smFISH) in mosaic clones of follicle cells (Figures 5A and S3). We observed a complete absence of the *flam* signal in *tj*-deficient mutant cells, whereas the *flam* signal at the nuclear periphery was detected in neighboring control cells that were Tj positive, consistent with previous reports.^53,54^ Furthermore, sequencing of small RNAs from ovaries with soma-specific knockdown (SKD) of *tj* (using a *tj*-Gal4 driver) showed a complete loss of piRNAs from the *flam* locus. (Figure 5B). Since Tj has been previously reported to regulate Piwi,^35^ and to rule out indirect effects, we investigated *flam* transcription by smFISH in follicle cells upon SKD of *piwi*. In contrast to *tj* mutant follicle cells, which lack *flam* expression, the absence of Piwi had no effect on *flam* levels (Figures 5C and 5D). In conclusion, our findings strongly support a key role of Tj in directly regulating the transcription of *flam* piRNA precursors in the somatic follicle cells of the adult ovary.

To investigate whether additional somatic TFs could be involved in the follicle cell-specific expression of *flam in vivo*, we carried out a translating ribosome affinity purification (TRAP) experiment. This method utilizes the UAS/Gal4 system and allows the identification of cell-specific mRNA translation.^55^ In adult *Drosophila* ovaries, we applied TRAP to the GFP-labeled follicle cell population using the *tj*-Gal4 driver (UASp-EGFP::RpL10A; *tj*-Gal4) and to the germ cell population with the *nos*-Gal4 driver (UASp-EGFP::RpL10A; *nos*-Gal4). Transcriptional profiling of purified mRNA followed by bioinformatic analyses identified around 900 genes with significantly higher expression and translation in somatic cells compared to germline cells (FC > 2, *p* < 0.05). In support of this, genes enriched in Tj-positive cells were associated with specific Gene Ontology biological processes related to the development of follicle somatic cells (Figure S4). Among the differentially enriched genes, 30 were DNA-binding factors, and this list included Tj (Table S2). Using *tj*-Gal4-mediated SKD, we next performed an RNAi screen targeting each candidate and retained only those that reproduced the *flam* mutant phenotype that is characterized by atrophied ovaries.^32,56^ We found that the depletion of *tj, Stat92E, schnurri, tango, chd64*, and *mamo* caused atrophic ovary development similar to *flam*. We next checked whether these factors affect *flam* expression by smFISH (Figure S5). Because the SKD of *Stat92E, tango, chd64, mamo*, and *schnurri* resulted in strongly atrophied ovaries, we instead transiently depleted these factors in adult follicle cells using a combination of the *tj*-Gal4 driver and the thermo-sensitive *tub*-Gal80ts repressor (*tub*-Gal80ts-*tj*-Gal4).^57^ A temperature shift from 18°C to 28°C enabled RNAi induction in late oogenesis stages, after ovaries had developed. In contrast to the atrophied ovary phenotype we observed with SKD, no impact on *flam* expression was detected following the depletion of *schnurri, tango, chd64*, and *mamo* using the thermo-sensitive RNAi approach (Figure S5). These results suggest that, while we cannot entirely rule out the contribution of multiple TFs, Tj appears to be the master TF responsible for the soma-specific expression of *flam*.

### Selective derepression of *gypsy*-family TEs following Tj depletion

**Figure 6:**
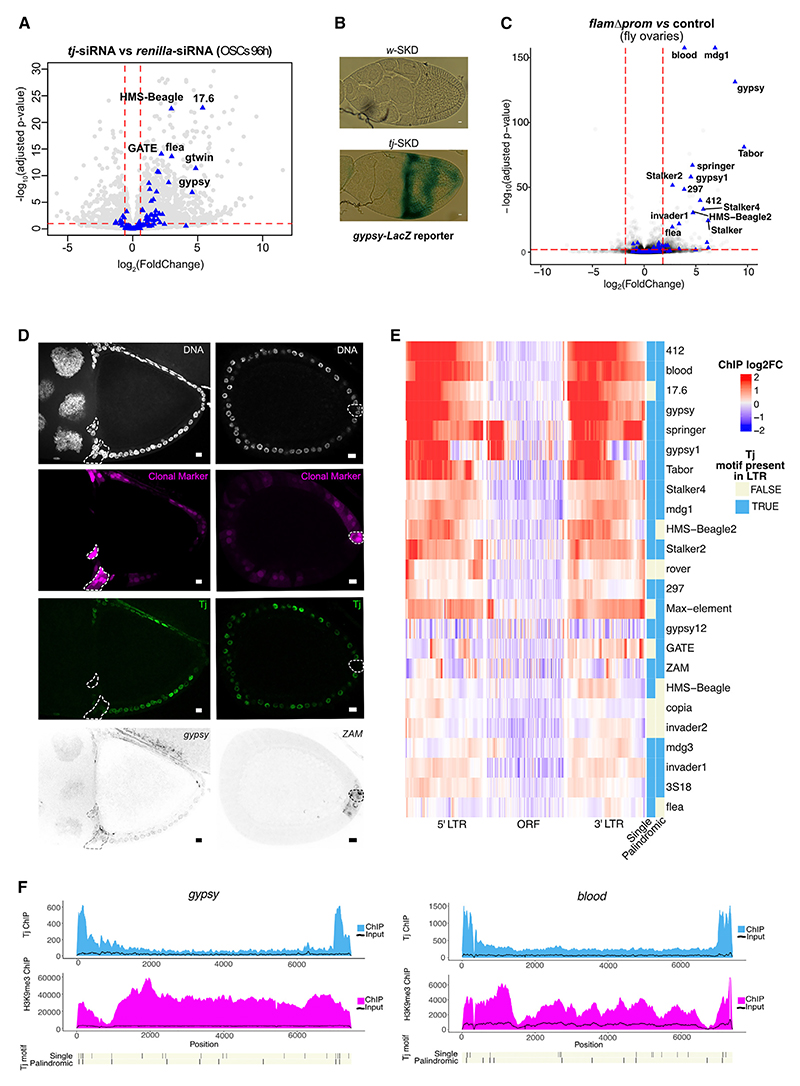
A subset of transposons appears to be directly regulated by Tj (A) Volcano plot showing upregulation of *gypsy-*family TEs by 96 h of *tj* siRNA knockdowns in OSCs using differential mRNA-seq analysis (DEseq2) between *tj* and *renilla* siRNA knockdowns (mRNA-seq, *n* = 3 replicates from distinct samples). Gray circles show the genes, and blue triangles show the transposons. (B) Shown are β-galactosidase (β-gal) stainings of egg chambers as readout for gypsy silencing in *tj*-SKD and w-SKD fly ovaries with *tj-Gal4*; *gypsy-LacZ* reporter. Scale bars: 10 μm. (C) Volcano plot showing upregulation of *gypsy-*family TEs in *flamΔprom* fly ovaries using differential mRNA-seq analysis between *flam* mutants (*flamΔprom homozygote*) and control (*flamΔprom heterozygote*) (DEseq2; *n* = 3 replicates from distinct samples). Gray circles show the genes and blue triangles show the transposons. TEs with *p*.adj. < 0.01 and >3.5 × fold change (FC) are considered differentially expressed. (D) Confocal images of *tj*-SKD-induced mosaic clonal egg chambers showing *gypsy* and *ZAM RNA* (gray), Tj (green), and clonal marker (magenta) by immuno-FISH. *tj*-SKD clones are circled in white. Nuclei are labeled with DAPI (white). Scale bars: 10 μm. (E) Heatmap showing log_2_FC of Tj ChIP-seq signal over input for the indicated TEs that were selected based on expression in *flamΔprom* vs. control fly ovaries or *tj* vs. control knockdown OSCs (*p*.adj. < 0.01 and baseMean normalized by TE length in the top 70% of values for each dataset). (F) Position plots showing the ChIP-seq and input signals and positions of single and palindromic Tj motifs along the TE consensus sequence for the *gypsy* (top) and *blood* (bottom).

Since *flam* and the somatic piRNA pathway factors control *gypsy*-family TEs in OSCs, we next asked whether Tj depletion resulted in transposon derepression. RT-qPCR revealed ~4- and ~20-fold upregulation of *gypsy* expression following 48 and 96 h of *tj* small interfering RNA (siRNA) treatment in OSCs, respectively (Figures S6A and S6B). RNA-seq from OSCs further confirmed the robust upregulation of *gypsy* and other *gypsy*-family TEs after 48 and 96 h of *tj* knockdown (Figures 6A and S6C). RT-qPCR from ovaries with *tj* SKD showed upregulation of *gypsy* and *ZAM* (Figure S6D). The upregulation of *gypsy* itself was validated using an established reporter (*tj*-Gal4; *gypsy*-LacZ) (Figure 6B). Surprisingly, although several *gypsy*-family TEs were derepressed in *flam* mutant ovaries (Figure 6C), only a subset of them were upregulated in ovaries or OSCs depleted of Tj. The TEs *mdg1* and *412*, which are upregulated in *flam* mutant ovaries (Figure 6C), remained repressed upon *tj* knockdown in OSCs or ovaries (Figures 6A and S6C).

As *tj*-SKD ovaries were strongly atrophied, we validated our results in *tj*-SKD-induced mosaic clones. Consistent with our RT-qPCR, smFISH detected derepression of *gypsy* and *ZAM*, but not *mdg1* or *412*, in *tj*-SKD-induced mosaic clones, compared to neighboring control cells (Figures 6D and S6E). We hypothesized that the differences in TE expression might be caused by some TEs also being directly regulated by Tj. Indeed, most of the TEs deregulated in *flamΔprom* flies, such as *412* and *mdg1*, showed strong Tj ChIP-seq peaks, particularly in the long terminal repeat (LTR) region (Figures 6E and 6F), which was distinct from the H3K9me3 signal for the same TE. Co-opted regulation by Tj could potentially explain why certain TEs are not derepressed following *tj* knockdown. In contrast, TEs such as *ZAM* and *297*, which show little to no Tj binding in their promoter regions, are upregulated under both *flamΔprom* and *tj*-knockdown conditions, consistent with their transcription being independent of direct Tj binding. In addition, we noted that some TEs, such as *gypsy*, display Tj binding yet remain derepressed upon *tj* SKD, suggesting that other somatic TFs could contribute to their expression or that residual Tj protein could be sufficient for their activation. Of note, this is supported by the accompanying study by Rivera and colleagues, who also showed that Tj binds and recognizes motifs within the LTRs of several TEs.^58^ Overall, these results suggest co-evolution between Tj-dependent regulatory regions of TEs and piRNA pathway genes and clusters.

## Discussion

Our study identifies Tj as a pivotal transcriptional regulator of the somatic piRNA pathway in *Drosophila* ovaries and adds a key layer to our understanding of TE repression mechanisms in *Drosophila*, particularly in non-germline tissues. Previous studies have demonstrated Tj’s involvement in gonadal development and its regulation of *piwi* expression.^34,35^ Our findings expand on these roles by demonstrating Tj’s broader control over somatic piRNA pathway components in ovarian follicle cells. Our results reveal Tj as a regulator of somatic piRNA pathway components, including *fs(1)Yb, nxf2, panx*, and *armi*. ChIP-seq and motif analyses demonstrate that Tj binding at conserved promoter motifs is critical for the expression of these genes. Moreover, Tj regulates the soma-expressed piRNA cluster *flam*, which is essential for TE silencing. This dual regulation of piRNA factors and clusters mirrors the function of mammalian TFs such as A-MYB, TCFL5, and BTBD18, which coordinate piRNA pathway gene expression and pachytene piRNA cluster activation during spermatogenesis.^59,60^

Beyond its role in piRNA regulation, Tj appears to be a master regulator of somatic cell identity, with approximately 8,000 Tj binding sites detected at the promoters of somatically enriched genes. RNA-seq analyses of *tj* knockdowns in OSCs revealed a significant shift from somatic to germline gene expression, with the upregulation of germline-specific genes such as *osk, ovo*, and *nos* (Table S1). This shift is accompanied by the downregulation of *l(3)mbt*, suggesting that Tj maintains the somatic expression program by repressing germline gene transcription. Interestingly, Tj and Ovo, a germline-specific TF,^33^ exhibit opposing binding patterns at the promoters of somatic and germline genes (Figure S7A), underscoring their complementary roles in maintaining lineage-specific gene expression programs within gonads (Figure S7B). However, while Ovo’s role in regulating germline piRNA factors is conserved across species, it is not clear whether Tj’s regulation of the somatic piRNA pathway is a *Drosophila*-specific adaptation or more broadly conserved. We speculate that this evolutionary innovation likely arose in response to the challenge posed by *gypsy*-family TEs, which can infect the germline via somatic expression.^61,62^ These TEs have evolved distinct expression patterns in somatic follicle cells.^61,62^ When the piRNA pathway is disrupted, some TEs are broadly expressed, while others remain restricted to specific niches, such as *ZAM* in posterior follicle cells and *Idefix* in the germarium, for example,^61–63^ indicating regulation by distinct TFs. Indeed, some TEs were reported to rely on specialized TFs, such as Pointed for *ZAM*,^64^ while our results indicate that other TEs are likely co-regulated by Tj, allowing for broader or more robust somatic expression. Notably, only a subset of TEs derepressed in *flam* mutants are also affected by Tj loss, suggesting that some TEs are independent of Tj for their expression. Co-evolution between TEs and the piRNA pathway could suggest an ongoing evolutionary “arms race” between host regulatory mechanisms and TEs. The coordinated expression of *flam* and somatic piRNA pathway components likely reflects the selective pressures exerted by TEs that shaped their *cis*-regulatory sequences to respond to one key TF in OSCs, allowing for the efficient and coordinated silencing of both niche-specific and broadly expressed TEs in somatic cells.

Although primarily considered a transcriptional activator, Tj may also function as a repressor—either at the transcriptional level by silencing adhesion molecule genes or post-transcriptionally through its 3′ UTR, which generates piRNAs that target and silence specific genes such as *FasIII*.^34,35^ The precise mechanisms by which Tj regulates its targets remain to be fully elucidated, particularly the potential involvement of additional transcriptional regulators and cofactors. Other factors such as Ci, Mirror, or Stat92E, which are all DNA-binding proteins expressed in somatic follicle cells that were identified in our TRAP-based screening, could contribute to the regulation of piRNA genes and *flam*, as is often described in gene regulatory networks where multiple TFs work together to ensure robustness and precise control across development.^65,66^

Tj’s similarity to mammalian Maf proteins, c-Maf and MafB in particular, including its potential for dimerization, adds another layer of specificity to its regulatory function. Like other members of this TF family, Tj contains a leucine zipper domain, a basic DNA-binding domain, and a Maf-specific extended homology domain in its C-terminal region.^34^ The leucine zipper domain, known for facilitating the dimerization of Maf proteins, either with other Maf or bZip TFs (e.g., homo/heterodimeric AP-1 complexes),^40^ suggests that Tj could also function as a dimer. The presence of pseudo-palindromic motifs within Tj binding sites, similar to the MARE motifs recognized by the dimeric Maf family proteins,^40,41^ further reinforces this idea. Interestingly, the pseudo-palindromic Tj motifs at promoters of the somatic piRNA factors were often mutated (Figure S1B), which has been previously shown to confer DNA-binding specificity for homodimeric bZip TF complexes over their heterodimeric versions in electrophoretic mobility shift assay (EMSA) experiments.^41^

In conclusion, our findings establish Tj as a master regulator of the somatic piRNA pathway and a key player in maintaining genome integrity through TE repression. By regulating both piRNA pathway components and the *flam* piRNA cluster, Tj exemplifies the efficiency of a single TF in orchestrating complex gene networks. Tj’s interaction partners, which likely contribute to its finely-tuned regulatory expression network, await further exploration.

### Limitations of the study

While our data suggest direct effects of Tj on the somatic piRNA pathway, we cannot entirely exclude the possibility of indirect effects. Tj regulates numerous somatic genes, including TFs, and its broad regulatory network may contribute indirectly to piRNA pathway activation. For example, the downregulation of *l(3) mbt*, a repressor of germline programs in somatic cells, observed upon *tj* knockdown could indirectly influence somatic piRNA pathway gene expression. Further investigation will be needed to disentangle these direct and indirect regulatory effects. Tj is known to regulate many genes critical for ovarian development, and therefore, the ovarian atrophy observed upon Tj loss could be a consequence of TE reactivation due to piRNA pathway disruption and/or misregulation of developmental genes critical for ovarian tissue homeostasis. In addition, our results with the *de novo* motif analysis with STREME did not yield the pseudo-palindromic motifs, likely due to a limitation in the detection of gapped motifs by the software as well as frequent mutations and the less frequent appearance of the pseudo-palindromes at specific gapped lengths (1–4 bp) within the peaks; however, we were able to map them using the motif enrichment (i.e., CentriMo in MEME-ChIP) and scanning (i.e., FIMO) tools.

### Resource Availability

#### Lead contact

Requests for further information and resources should be directed to and will be fulfilled by the lead contact, Emilie Brasset (emilie.brasset@uca.fr).

#### Materials availability

All unique/stable reagents generated in this study are available from the lead contact with a completed materials transfer agreement.

## Star★Methods

Detailed methods are provided in the online version of this paper and include the following:

- KEY RESOURCES TABLE
- EXPERIMENTAL MODEL AND STUDY PARTICIPANT DETAILS
  ○ D. melanogaster flies
  Wild-type OSCs
- METHOD DETAILS
  ○ Cell culture and growth media
  ○ siRNA knockdowns in cell culture
  ○ RNA isolation and RT-qPCR
  ○ RNA-seq
  ○ Fly stocks and crosses
  ○ Clones and constructs
  ○ Transgenic animal production
  ○ Generation of *flam* knockout line by CRISPR/Cas9
  ○ Mitotic clones of *tj* mutant or knockdown analysis
  ○ β-Gal staining on ovarioles
  ○ FISH and immunostaining
  ○ Immunofluorescence
  ○ TRAP experiment and RNA sequencing analysis
  ○ Small RNA sequencing
  ○ Small RNA-seq analysis
  ○ ATAC-seq data analysis
  ○ RNA-seq data analysis
  ○ ChIP-seq data analysis
  ○ Motif discovery and ATAC-seq peak conservation
- QUANTIFICATION AND STATISTICAL ANALYSIS

## Star★Methods

### Key Resources Table

**Table:** 

REAGENT or RESOURCE	SOURCE	IDENTIFIER
Antibodies		
Goat Polyclonal anti-Armitage (dD-17) antibody	Santa Cruz Biotechnology	Cat# SC-34564; RRID: AB_1134112
Chicken polyclonal anti-mCherry antibody	NOVUS	Cat# NBP2-25158; RRID: AB_2636881
Guinea pig polyclonal anti-Traffic jam antibody	Gift form Dorothea Godt Lab	N/A
Rabbit polyclonal anti-Traffic jam	Gift from Nelson Lau lab	N/A
Cy™3 AffiniPure Donkey Anti-Goat IgG (H + L)	Jackson ImmunoResearch Labs	Cat# 705-165-147; RRID: AB_2307351
Cy3-AffiniPure Donkey Anti-Chicken IgY (IgG) (H + L)	Jackson ImmunoResearch Labs	Cat# 703-165-155; RRID: AB_2340363
Alexa Fluor 488-AffiniPure Donkey Anti-Guinea Pig IgG (H + L)	Jackson ImmunoResearch Labs	Cat# 706-545-148; RRID: AB_2340472
Cy3-AffiniPure Donkey Anti-Rat IgG (H + L)	Jackson ImmunoResearch Labs	Cat# 712-165-150; RRID:AB_2340666
Biological samples		
Fly extract	*Drosophila* Genomics Resource Center (DGRC)	Cat#1645670
Fetal bovine serum	Sigma-Aldrich	Cat#F9665-500ML
Chemicals, peptides, and recombinant proteins		
Formaldehyde solution 16%	Polysciences	Cat#18814_10
Triton X-100	euromedex	Cat#2000_B
Formamide	Sigma-Aldrich	Cat#F7503
Dextran sulfate	Sigma-aldrich	Cat#9011-18-1
Atto-488	Lumiprobe	Cat#41698
Atto 550	Sigma-aldrich	Cat#92835
SSC20X	euromedex	Cat#EU0300-C
VectaShield	Vector Laboratory	Cat#H1000
BSA	euromedex	Cat#04-100-812-E
TRIzol Reagent	invitrogen	Cat#15596026
Shields and Sang M3 Insect Medium	Sigma-Aldrich	Cat#S3652-6X1L
Glutathione	Sigma-Aldrich	Cat#G6013-25G
Human insulin	Sigma-Aldrich	Cat#I9278-5ML
Penicillin-Streptomycin	Gibco	Cat#15070063
Critical commercial assays		
NEB Gibson Assembly Cloning Kit	New England Biolabs	Cat#E5510
QuickExtractTM DNA Extraction Solution	Cambio	Cat#QE0905T
SuperScript IV reverse transcriptase	Invitrogen	Cat#18090010
RQ1 RNase-Free DNase	Promega	Cat#M6101
LightCycler 480 Green Master	Roche Diagnostic	Cat#4887352001
Nucleofection solution V	Lonza	Cat#VVCA-1003
Deposited data		
Small RNA-seq and mRNA-seq	This paper	GSE282040
Experimental models: Cell lines		
Ovarian somatic cells (OSCs)	*Drosophila* Genomics Resource Center (DGRC)	Stock # 288
Experimental models: Organisms/strains		
*D. melanogaster:* line expressing dsRNA for RNAi of ***w*** under UAS control in the VALIUM20 vector. y[1] v[1]; P{y[+t7.7] v[+t1.8] = TRiP.HMS00045}attP2	Bloomington Drosophila Stock Center	Flybase: FBgn0003996; RRID:BDSC_33644
*D. melanogaster:* line expressing dsRNA for RNAi of ***tj*** under UAS control in the VALIUM10 vector. y[1] v[1]; P{y[+t7.7] v[+t1.8] = TRiP.JF02009}attP2	Bloomington Drosophila Stock Center	Flybase:FBgn0000964; RRID:BDSC_25987
*D. melanogaster:* line expressing dsRNA for RNAi of ***tj*** under UAS control in the VALIUM10 vector. y[1] sc[*] v[1] sev[21]; P{y[+t7.7] v[+t1.8] = TRiP.HMS01069}attP2	Bloomington Drosophila Stock Center	Flybase: FBgn0000964; RRID:BDSC_ 34595
*D. melanogaster:* line expressing dsRNA for RNAi of ***Stat92E*** under UAS control in the VALIUM22 vector. y[1] sc[*] v[1] sev[21]; P{y[+t7.7] v[+t1.8] = TRiP.GL00437}attP40/CyO	Bloomington Drosophila Stock Center	Flybase: FBgn0016917; RRID:BDSC_35600
*D. melanogaster:* line expressing dsRNA for RNAi of ***tgo*** under UAS control in the VALIUM20 vector. y[1] sc[*] v[1] sev[21]; P{y[+t7.7] v[+t1.8] = TRiP.HMC03580}attP40	Bloomington Drosophila Stock Center	Flybase: FBgn0264075; RRID:BDSC_53351
*D. melanogaster:* line expressing dsRNA for RNAi of *Chd64* under UAS control in the VALIUM20 vector. y[1] v[1]; P{y[+t7.7] v[+t1.8] = TRiP. HMJ23773}attP40/CyO	Bloomington Drosophila Stock Center	Flybase: FBgn0035499; RRID:BDSC_62377
*D. melanogaster:* line expressing dsRNA for RNAi of ***mamo*** under UAS control in the VALIUM20 vector. y[1] sc[*] v[1] sev[21]; P{y[+t7.7] v[+t1.8] = TRiP.HMC05105}attP2	Bloomington Drosophila Stock Center	Flybase: FBgn0267033; RRID:BDSC_60111
*D. melanogaster:* line expressing dsRNA for RNAi of ***shn*** under UAS control in the VALIUM20 vector. y[1] sc[*] v[1] sev[21]; P{y[+t7.7] v[+t1.8] = TRiP.HMS06091}attP40	Bloomington Drosophila Stock Center	Flybase: FBgn0003396; RRID:BDSC_82982
*D. melanogaster:* line expressing dsRNA for RNAi of ***piwi*** under UAS control in the VALIUM20 vector. y[1] sc[*] v[1] sev[21]; P{y[+t7.7] v[+t1.8] = TRiP.HMS00606}attP2	Bloomington Drosophila Stock Center	Flybase: FBgn0004872; RRID:BDSC_33724
*D. melanogaster:* line *tj*-Gal4 driver Expresses GAL4 under the control of the traffic jam promoter. y[*] w[*]; P{w[+mW.hs] = GawB}NP1624/CyO, P{w[-] = UAS-lacZ.UW14}UW14	Kyoto Stock Center (DGRC	Flybase: FBti0034540; RRID:DGGR_104055
*D. melanogaster:* line *tub*-Gal80ts-*ty*-Gal4 driver Expresses temperature-sensitive GAL80 under the control of the alphaTub84B promoter. Restrictive temp is 30°C. y[*] w[*]; *P{tubP-GAL80[ts]}10,* P{w[+mW.hs] = GawB}NP1624/CyO, P{w[-] = UAS-lacZ.UW14}UW14	Construction realized by Mirouse Lab, Dennis et al.^67^ Recombination between DGGR#10455 and BDSC#67059	FlyBase: FBti0027796; RRID:BDSC_67059 Flybase: FBti0034540; RRID:DGGR_104055
*D. melanogaster:* line hsFlp; FRT40A-nls-RFP y,w, hs:FLP: P{ry[+t7.2] = hsFLP}22, w[*]; P{w[+mC] = Ubi-GFP(S65T)nls}2L P{ry[+t7.2] = neoFRT}40A/CyO	Construction realized by Mirouse Lab, Dennis et al.^67^ between BDSC#8862 and BDSC#5629. Bloomington Drosophila Stock Center	FlyBase ID: FBst0008862; BDSC: 8862 FlyBase ID: FBti0015576; BDSC: 5629
*D. melanogaster:* line b tjeo2 FRT40A/CyO, bw	from Dorothea Godt lab, University of Toronto	
*D. melanogaster: hs-hid(Y); tj Gal4, gypsy_lacZ/CyO;;*	Vienna BioCenter Core Facilities (VDRC)	VDRC: 313222
*D. melanogaster: y,w,HS:flp122/+;* *Act:FRTstopFRTGal4, UAS:GFP/CyO;*	Vialat et al.^68^	
*D. melanogaster:* line expressing TdTomato under the control of *flam[−515; +30] Chr2, 53B2, 2R:16298254 flam[−515; +30]tdTomato-K10 polyA*	This paper	N/A
*D. melanogaster*: line expressing *TdTomato* under the control of *flam[−515; +432] Chr2, 53B2, 2R:16298254 flam[−515; +432]TdTomato-K10 polyA*	This paper	N/A
*D. melanogaster*: line expressing *TdTomato* under the control of *flam[−515; +718] Chr2, 53B2, 2R:16298254 flam[−515; +718]TdTomato-K10 polyA*	This paper	N/A
*D. melanogaster*: line expressing *TdTomato* under the control of *flam[−515; +1200] Chr2, 53B2, 2R:16298254 flam[−515; +1200]TdTomato-K10 polyA*	This paper	N/A
*D. melanogaster*: line expressing *TdTomato* under the control of *flam[−515; +2086] Chr2, 53B2, 2R:16298254 flam[−515; +2086]TdTomato-K10 polyA*	This paper	N/A
*D. melanogaster*: line expressing *TdTomato under the control offlam[−515; +6334] Chr2, 53B2, 2R:16298254 flam[−515; +6334]TdTomato-K10 polyA*	This paper	N/A
*D. melanogaster*: line expressing *TdTomato* under the control of *flam[-1626; +718] Chr2, 53B2, 2R:16298254 flam[-1626; +718]TdTomato-K10 polyA*	This paper	N/A
*D. melanogaster*: line expressing *TdTomato* under the control of *flam[-1626; +2086] Chr2, 53B2, 2R:16298254 flam[-1626; +2086]TdTomato-K10 polyA*	This paper	N/A
*D. melanogaster*: line expressing *TdTomato* under the control of *flam[-770; +2086] Chr2, 53B2, 2R:16298254 flam[+770; +2086]TdTomato-K10 polyA*	This paper	N/A
*D. melanogaster*: line expressing *TdTomato* under the control of *14XUAS flam[−515; +432] Chr2, 53B2, 2R:16298254 14xUAS flam[−515; +432]TdTomato-K10 polyA.*	This paper	N/A
*D. melanogaster:* line with a deletion of ChrX:21631436-21632105 in the promoter of *flam*	This paper	N/A
Oligonucleotides		
Probes for *in situ* hybridization, see Table S3	This paper	N/A
Primers for plasmids construction, see Table S3	This paper	N/A
sgRNA see, Table S3	This paper	N/A
Primers used to check *flam* mutant lines, see Table S3	This paper	N/A
RT-qPCR primers, see Table S3	This paper	N/A
Recombinant DNA		
Plasmid constructions listed see on Table S3	This paper	N/A
pCFD5	Addgene	Cat#73914
W + AttB	Addgene	Cat#30326
Software and algorithms		
FastQC (fastqcr_0.1.3)	https://www.bioinformatics.babraham.ac.uk/ projects/fastqc/	
Bowtie2 (version 2.4.2)	Langmead et al.^69^	
HTSeq count	Anders et al.^70^	
Features count (v2.0.1)	Liao et al.^71^	
DESeq2 (DESeq2_1.30.1)	Love et al.^72^	
Bioconductor	Yu et al.^73^	
R version 4.0.4	https://www.r-project.org	
sRNAPipe	Pogorelcnik et al.^74^	
Bedtools	https://bedtools.readthedocs.io/en/latest/	
Omero	https://www.openmicroscopy.org/omero/	
UCSC Genome Browser	https://genome.ucsc.edu/	
UCSC LiftOver tool	https://genome.ucsc.edu/cgi-bin/hgLiftOver	
FIMO tool in MEME Suite (v5.4.1)	https://meme-suite.org/meme/tools/fimo	
MEME- ChIP tool in MEME Suite (v5.4.1)	https://meme-suite.org/meme/tools/meme-chip	
CentriMo in MEME Suite (v5.4.1)	https://meme-suite.org/meme/tools/centrimo	
UCSC Table Browser	https://genome.ucsc.edu/cgi-bin/hgTables	
Burrows-Wheeler Aligner (BWA) (v0.7.17)		
SAMtools (v1.9)		
MACS2 (v2.1.1.20160309)		
deepTools (v3.5.1)	https://deeptools.readthedocs.io/en/3.5.1/	
STAR (v2.7.3a) and v(2.7.8a)		
featureCounts tool (Subread package v1.5.3)	https://subread.sourceforge.net/	
MACS3 (v3.0.0a6)		
irreproducible discovery rate (IDR) tool (v2.0.2)		
Cutadapt tool (v1.18)		
Picard tool (v2.9.0)	https://broadinstitute.github.io/picard/	
bedtools	https://bedtools.readthedocs.io/en/latest/	
ataqv (v1.0.0)	https://github.com/ParkerLab/ataqv	
DSIR tool	http://biodev.cea.fr/DSIR/DSIR.html	
Other		
Reanalyzed Adult ovary soma vs. germline TRAP experiment see Table S4	Vachias et al.^75^	GSE230452
Reanalyzed ChIP seq pol2 in ovaries see Table S4	Andersen et al.^20^	GSE97719
Reanalyzed ChIP-seq data of Input control for Tj ChIP-seq in female see Table S4	Kudron et al.^39^	ENCSR414VZP
Reanalyzed ChIP seq data of Tj binding in female whole fly see Table S4	Kudron et al.^39^	ENCSR199TFG
Reanalyzed ChIP seq data of Input and H3K9me3 in siGFP OSCs see Table S4	Eastwood et al.^16^	GSE160855
Reanalyzed ATAC-seq data of OSC and *Drosophila melanogaster* whole ovary see Table S4	Alizada et al.^33^	GSE233246
Reanalyzed ATAC-seq data of *Drosophila yakuba* whole ovary see Table S4	van Lopik et al.^30^	GSE225889
Reanalysed RNA-seq data of OSCs, ovarian germ cells, and ovarian somatic cells see Table S4	Alizada et al.^33^	GSE233246
Reanalysed RNA-seq data of *Drosophila melanogaster* and *Drosophila yakuba* whole ovary see Table S4	van Lopik et al.^30^	GSE225889
Fly TF motifs	FlyFactorSurvey (http://pgfe.umassmed.edu/TFDBS/)	
Vertebrate TF motifs	JASPAR2022_CORE_vertebrates_ non-redundant, EUKARYOTE/ jolma2013, and MOUSE/ uniprobe_mouse motif databases	
*D. melanogaster* gene annotations	Ensembl genome database	Drosophila_melanogaster. BDGP6.28.100.gtf
Orthologous *D. yakuba* genes	Genes from chained tBLASTn in UCSC Table Browser	blastDm2FB
Consensus *Drosophila* transposon sequences	https://github.com/bergmanlab/transposons	

### Experimental Model And Study Participant Details

#### D. melanogaster flies

Genotypes: listed in Key resources table.

Age: Adult stages, 3 to 6 days old.

Sex: female (to study ovaries)

Housing: 20°C or 25°C, on standard medium.

For heat shock experiments flies are placed 1h at 37°C on standard Medium.

#### Wild-type OSCs

Species: D. melanogaster

Sex: female.

Tissue: ovary.

Growth media: cultured at 26°C in Shields and Sang M3 Insect Medium (Sigma-Aldrich, S3652-6X1L) supplemented with 10% fetal bovine serum (Sigma-Aldrich, F9665-500ML), 10% fly extract (DGRC, Stock # 1645670), 0.6 mg/mL glutathione (Sigma-Aldrich, G6013-25G), and 10 mU/mL human insulin (Sigma-Aldrich, I9278-5ML) + 1% Penicillin-Streptomycin (Gibco, 15070063).

### Method Details

#### Cell culture and growth media

Wild-type OSCs^33^ were purchased from the *Drosophila* Genomics Resource Center (DGRC, cat # 288) and cultured at 26°C in Shields and Sang M3 Insect Medium (Sigma-Aldrich, cat #S3652-6X1L) supplemented with 10% fetal bovine serum (Sigma-Aldrich, cat #F9665-500ML), 10% fly extract (DGRC, cat # 1645670), 0.6 mg/mL glutathione (Sigma-Aldrich, cat #G6013-25G), 10 mU/mL human insulin (Sigma-Aldrich, cat #I9278-5ML), and 1% Penicillin-Streptomycin (Gibco, cat # 15070063).

#### siRNA knockdowns in cell culture

Sense and antisense 21 nt siRNA sequences for target genes were designed using the DSIR tool (http://biodev.cea.fr/DSIR/DSIR.html) (Table S3) and ordered from IDT. The RNA oligonucleotides were resuspended to 400 mM final concentration in RNase-free water. Equal volumes of the resuspended sense and antisense siRNA were mixed and then added to an equal amount of 2x annealing buffer (60 mM potassium acetate; 200 mM HEPES, pH 7.5). The mix was boiled for 5 min at 75°C, then ramped down to 25°C (-0.1 °C/s) to anneal the siRNA sequences into the siRNA duplex (100 mM final concentration). 2 mL of the final 100 mM siRNA duplex was mixed with 100 mL of Nucleofection solution V (Lonza, VVCA-1003) and transfected into 4 million cells (48 h knockdown) or 5 mL of 100 mM siRNA duplex was mixed with 100 mL of Nucleofection solution V and transfected into 10 million cells (96 h knock-down) using a NucleofectorTM 2b Device and program T-029 (Lonza). Cells were plated into 6-well plates (5 million cells) or 10 cm plates (10 million cells). RNA was either harvested at 48 h or nucleofection was repeated after 48 h and RNA was harvested at 96 h. RNA was either harvested at 48 h or nucleofection was repeated after 48 h and RNA was harvested at 96 h.

#### RNA isolation and RT-qPCR

OSCs were homogenized by addition of Buffer RLT directly on the cell culture vessel surface. RNA was isolated using RNeasy Mini Kit (QIAGEN, 74106) with RNase-Free DNase Set (QIAGEN, 79254) for DNase digestion during RNA purification as per manufacturer’s instruction. Reverse transcription was performed with 100 ng to 1 μg RNA using SuperScript IV Reverse Transcriptase (Thermo Fisher Scientific, 18090010). RT-qPCR was performed on 1:10 diluted cDNA using Fast SYBR Green Master Mix (Thermo Fisher Scientific, 4385610) with Bio-Rad C1000 Thermal Cycler. Primers were designed against exon-exon junctions for genes with introns and *rpL32* was used as an internal standard (Table S3). Relative expression was analyzed using the delta-delta Ct method.^76^

When total RNAs were extracted from fresh ovaries, we used TRIzol (Invitrogen) following the manufacturer’s instructions. After DNase treatment (RQ1 RNase-free DNase Promega), cDNA was synthesized using random priming of 1 μg total RNA and SuperScript IV Reverse Transcriptase (Invitrogen). Quantitative PCR was performed with the LightCycler480 SYBR Green I Master mix system (Roche), and the data were analyzed using the LightCycler480 system (Roche). Each experiment was performed 6 times with technical duplicates. RNA levels were calculated with the relative quantification method with *rpL32* housekeeping gene as the reference (for primer sequences, see Table S3). Statistical differences were evaluated using Wilcoxon-Mann-Whitney non-parametric test, with corrections for multiple comparisons (Holm–Bonferroni adjustment method).

For mRNA-seq experiments, total RNA was extracted from 30 ovaries using TRIzol (Invitrogen) following the manufacturer’s instructions.

#### RNA-seq

For mRNA-seq from OSCs, selection was performed using Poly(A) mRNA Magnetic Isolation Module (NEB, E7490) with 1 μg of total RNA as input. Libraries were prepared using The NEBNext Ultra Directional RNA Library Prep Kit for Illumina (NEB, E7420L). Library quality was assessed using Agilent 2100 bioanalyzer (High Sensitivity DNA Kit). Libraries were quantified with KAPA Library Quantification Kit for Illumina (Kapa Biosystems, cat# KK4873) submitted to sequencing on a NovaSeq X System with 2 ×50 bp reads.

For mRNA-seq from ovaries 1 μg of total RNA was send to BGI for library construction using the DNBSEQ Eukaryotic Strand-specific mRNA library workflow. Libraries were sequenced on platform DNBSEQ to a read length of 150 bp paired-end. Raw data were filtered by BGI in order to remove adapter sequences or low-quality reads.

#### Fly stocks and crosses

All *D. melanogaster* stocks were grown on standard medium at 20 C. RNAi lines expressing dsRNAs^80^ or shRNAs^81^ against *w* (#33644), *tj* (#25987, #34595), *Stat92E* (#35600), *tango* (#53351), *chd64* (#62377), *mamo* (#60111), *schnurri (#82982), piwi* (#33724) were obtained from the Bloomington *Drosophila* stock center (BDSC). The *tj*-Gal4 driver was originated from Kyoto Stock Center DGRC, #104055. The *gypsy*-lacZ strain (#313222) was obtained from Vienna *Drosophila* Resource Center (VDRC). The lines *tub-*Gal80ts-*tj*-Gal4 driver and hsFlp; FRT40A-nls-RFP were a kind gift of V. Mirouse at iGReD^67^ and the *tj*^*eo2*^ FRT40A/CyO, bw line from Dorothea Godt (University of Toronto). The line *y*,*w*,*HS:flp122/+;Act:FRTstopFRTGal4, UAS:GFP/CyO;* is a kind gift of C. De Joussineau. The *tj*-Gal4 driver strain was crossed with the transgenic 14xUAS *flam*[−515; +482] *tomato* flies and with all RNAi strains. *tub-Gal80ts-tj-Gal4* line was crossed with RNAi lines.

#### Clones and constructs

All plasmids were constructed using NEB Gibson Assembly Cloning Kit (cat #E5510) in accordance with the manufacturer’s instructions and the recommendations of the NEBuilder assembly tool (https://nebuilder.neb.com/). Inserts (listed in the Table S3) were amplified by PCR using Phusion taq polymerase (Thermofisher) using primers summarized in Table S3, and cloned in the W + attB plasmid (addgene #30326). Ten vectors have been then elaborated in W + attB backbone: nine of them possessed different *flam* fragments sizes upstream of tdTomato reporter sequence directly followed by polyA signal sequence from *K10* gene:

*flam[−515; +30]tdTomato -K10 polyA, flam[−515; +432]tdTomato -K10 polyA, flam[−515; +718]tdTomato -K10 polyA, flam [−515; +1200]tdTomato -K10 polyA, flam[−515; +2086]tdTomato -K10 polyA, flam[−515; +6334]tdTomato -K10 polyA, flam [-1626; +718]tdTomato -K10 polyA, flam[-1626; +2086]tdTomato-K10 polyA, flam[+770; +2086]tdTomato-K10 polyA*. The last construct contains the fourteen UAS sequence upstream of the *flam[−515; +432]tdTomato-K10 polyA fragment and is named 14xUAS flam[−515; +432]tdTomato-K10 polyA*.

#### Transgenic animal production

Plasmid constructions were purified using Plasmid Plus Maxi Kit (QIAGEN). Purified DNA was used for PhiC31 integrase-mediated transgenesis, which was carried out by BestGene (http://www.thebestgene.com/). Transgenes were integrated into the genomic cytological position 53B2.

#### Generation of *flam* knockout line by CRISPR/Cas9

To remove the entire promoter region of *flamenco* we decided to delete the specific locus ChrX:21631436–21632105 (−455; +215 around TSS). To do so, we used two flanking sgRNAs. The two gRNAs were designed using CRISPR optimal target finder.^82^ The individual guide sequences are listed in the Table S3. Bolded sequences correspond to each sgRNA. Those primers were used on a pCFD5 template. The resulting PCR product was assembled with the linearised pCFD5 backbone in a single Gibson Assembly reaction. Then the plasmid was injected in *vas*-Cas9 embryos (BestGene, BL#51324). To establish KO lines, molecular characterisation of target loci was performed as described.^77^ Briefly, genomic DNA was extracted from individual larvae using QuickExtract DNA Extraction Solution (Cambio) according to the supplier’s instructions. Mutant flies were selected by PCR reactions. Exact deletion of *flam* was precisely identified by PCR and sanger sequencing once homozygote mutant fly lines were obtained (primers listed in Table S3).

#### Mitotic clones of *tj* mutant or knockdown analysis

For mitotic clones of tj mutant, the *Drosophila* strain *hsFlp; FRT40A - nls-RFP*; was crossed with the *tjeo2 FRT40A/CyO, bw* strain. For mitotic clones of *tj* knockdown, *y*,*w*,*HS:flp122/+;Act:FRTstopFRTGal4, UAS:GFP/CyO;* flies were crossed with lines carrying shRNAs against *tj* or *w*. All these flies were maintained at 25°C on a rich medium. Three heat shocks at 37°C for 1 h were applied to the F1 progeny, one day before eclosion (pupal stage), on the day of eclosion and one day after. The following day, ovaries were dissected and fixed for either FISH or IF experiments.

#### b-Gal staining on ovarioles

Ovaries from 5-day-old flies were dissected in PBS, kept on ice, fixed in 0.2% glutaraldehyde/2% formaldehyde/PBS at room temperature for 5 min and rinsed three times with PBS. They were then incubated in staining solution (1x PBS pH7.5, 1mM MgCl2, 4mM potassium ferricyanide, 4mM potassium ferrocyanide, 1% Triton, 2.7 mg/ml X-Gal) at 37°C for 1h.^78^

#### FISH and immunostaining

Five to ten ovary pairs were dissected into ice-cold PBS and fixed in formaldehyde solution (4% formaldehyde, 0.3% Triton X-100 in PBS) for 20 min at RT with agitation. The fixed ovaries were then washed three times for 10 min in 0.3% Triton X-100/PBS and permeabilized overnight at 4°C in 70% ethanol. For probe hybridization, permeabilized ovaries were first rehydrated for 5 min in RNA FISH wash buffer (10% (v/w) formamide in 2x SSC). The ovaries were then resuspended in 50 mL hybridization buffer (10% (v/w) dextran sulfate and 10% (v/w) formamide in 2x SSC). Subsequently, 1.5 mL of a homemade *flam* RNA probe labeled with Atto-488 (Sigma, ref. 41698) or Atto-550 (Lumiprobe, ref. 92835) was added with or without primary antibody. The samples were incubated at 37°C with agitation. The ovaries were then rinsed twice for 15 min at RT in RNA FISH wash buffer. For FISH-IF, incubation with a secondary antibody conjugated to either Alexa 488, Cy3, or Cy5, was performed in 2x SSC for 1 h 30 at room temperature. After two washes in 2x SSC, DNA was stained with DAPI (1/500). The tissues were then mounted between a slide and coverslip using VectaShield mounting medium (Vector laboratories).

#### Immunofluorescence

Ovaries from 3-to 5-day-old flies were dissected in Schneider’s *Drosophila* Medium, fixed in 4% formaldehyde/PBS for 20 min, rinsed three times with PBT (×1 PBS, 0.1% Triton, 1% BSA), and incubated in PBT for at least 1 h 30. They were then incubated with primary antibodies overnight. After 3 washes in PBT, ovaries were incubated with the corresponding secondary antibodies (1:1,000), conjugated to Alexa 488, Cy3, or Cy5, respectively, for 90 min. After two washes, DNA was stained with DAPI. The tissues were then mounted between a slide and coverslip using VectaShield mounting medium (Vector laboratories). All antibodies used for immunostaining (IF) along with probe sequences for *in situ* hybridization (FISH) are listed in Table S3. FISH and IF experiments were imaged using Zeiss LSM 800 or LSM 980 Airyscan confocal with a 20x and 40×objectives and analyzed using OMERO figure software.

#### TRAP experiment and RNA sequencing analysis

The TRAP experiment was described by Vachias and colleagues^75^ and data was publicly available (see Table S4). Quality control of the sequencing data was evaluated using FastQC software. High quality reads were mapped to the *Drosophila melanogaster* dm6 reference genome using bowtie2 with default parameters.^69^ Reads per gene were counted using HTSeqcount.^70^ Normalization and differential gene expression analysis were performed using DESeq2.^72^ Only genes with an adjusted *p*-value <0.05 were considered as differentially expressed between the two conditions. Gene expression data from Tj positive cells were compared to Nanos dataset by applying a fold change ≥2, *p* < 0.05 in order to generate list of enriched somatic genes. We computed and compared GO biological processes and molecular functions from this list using an R package cluster profiler from BioConductor.^73^

#### Small RNA sequencing

Total RNA was extracted from 80 to 100 pairs of ovaries from 3-to 5-day-old flies (for the analysis of piRNA production in somatic follicle cells) using TRIzol reagent (Invitrogen). After 2S rRNA depletion, deep sequencing of 18–30-nt small RNAs was performed by Fasteris S.A. (Geneva/CH) (Table S4).

#### Small RNA-seq analysis

Illumina small RNA-Seq reads were analyzed with the small RNA pipeline sRNAPipe^74^ to map them to various genomic sequence categories of the *D. melanogaster* genome (release 6.03). All libraries were normalized per million of either genome-mapped reads or unique genome-mapping piRNAs (0 mismatches). All the analyses were performed using genome-unique piRNAs mapped to piRNA clusters, as defined by Brennecke and colleagues^6^ (no mismatch allowed). The size profile for *flam* piRNA cluster was obtained by extracting the reads of 23–29 nt sequences uniquely mapped to this locus. Subsequent quantification of reads mapping to 1-kb tiles was done using bedtools, while relative quantification and plotting were done in R. Briefly, small RNA-seq tile signal was normalized to the estimated mappability scores for each 1-kb window. A pseudo-count of 1 was then added to each tile value and log2(fold-change) values relative to control samples was performed.

#### ATAC-seq data analysis

The read quality was assessed with FastQC (v0.11.8). The paired-end reads were trimmed of adapter sequences TCGTCGGCAG CGTCAGATGTGTATAAGAGACAG and GTCTCGTGGGCTCGGAGATGTGTATAAGAGACAG using Cutadapt tool (v1.18; default parameters).^79^ Burrows-Wheeler Aligner (BWA) (v0.7.17, bwa mem -M -t 4)^80^ was used to align the trimmed paired reads to 2014 (BDGP Release 6 + ISO1 MT/dm6) assembly of the *D. melanogaster* genome. Duplicates were marked using Picard tool (v2.9.0) (MarkDuplicates, validation stringency = lenient). SAMtools (v1.9) was used for indexing and filtering.^80^ The quality metrics for the aligned ATAC-seq reads were assessed using ataqv (v1.0.0) (https://github.com/ParkerLab/ataqv).^81^ The ATAC-seq peaks were called with MACS2 (v2.1.1.20160309) using –nomodel –shift −37 –extsize 73 parameters and FDR cut-off q ≤ 0.05^82^. RPKM normalized bigWig files were generated using deepTools (v3.5.1, bamCoverage).^83^ Data were visualized using the UCSC Genome Browser.^84^

#### RNA-seq data analysis

The reads were trimmed of adapter sequences using Cutadapt tool (v1.18; -m 1 specified to not have reads trimmed to zero).^79^ The trimmed reads were aligned to the genome assemblies using the RNA-seq aligner STAR (v2.7.3a).^85^ Gene counts were calculated with the featureCounts tool (Subread package v1.5.3; -s 2 for stranded libraries prepared by dUTP method).^71^ *D. melanogaster* gene annotations were taken from Drosophila_melanogaster.BDGP6.28.100.gtf in the Ensembl genome database^86^ and annotations of orthologous *D. yakuba* genes were derived from *D. melanogaster* proteins mapped onto *D. yakuba* genome by chained tBLASTn (blastDm2FB in UCSC Table Browser). The read depth-normalized ovary RNA-seq counts were then normalized to species gene lengths. Consensus *Drosophila* transposon sequences were taken from (https://github.com/bergmanlab/transposons). Samtools (v1.9) was used to merge bam files from replicates.^80^ Differential gene expression analysis was performed using DESeq2 (v1.30.1)^72^. The RPKM normalized bigWig files were generated for each strand using bamCoverage tool (–filterRNAstrand specified for dUTP stranded libraries) in deepTools (v3.5.1)^83^. Raw data for publicly available RNA-seq reads were downloaded from sources listed in (Table S4) and processed as above. Data were visualized using the UCSC Genome Browser.^84^

After quality control of reads using FASTQC (fastqcr_0.1.3), high quality reads were mapped to the *Drosophila melanogaster* dm6 reference genome (release 6_48 from flybase database), and consensus *Drosophila* transposon sequences (https://github.com/bergmanlab/transposons) using STAR (v2.7.8a).^85^ Gene counts were calculated with the featureCounts tool (featureCounts v2.0.1). Normalization and differential gene expression analysis were performed using DESeq2^72^ (DESeq2_1.30.1). Volcano plots were generated using ggplot2 (v 3.3.3) in R (v 4.0.4). To consider a gene or transposon to be differentially expressed, we required a 3.5-fold change and padj<0.01 unle.

#### ChIP-seq data analysis

The reads were trimmed of adapter sequences using Cutadapt tool (v1.18).^79^ The trimmed reads were aligned to the genome assemblies and TE consensus sequence assemblies (https://github.com/bergmanlab/drosophila-transposons/) using the Burrows-Wheeler Aligner (BWA) (v0.7.17, bwa aln).^87^ SAMtools (v1.9) was used for sorting, merging, and indexing.^80^ ChIP-seq peaks were called using MACS3 (v3.0.0a6) callpeak command (-q 0.01)^82^ with data from ChIP input (the whole cell lysate) used as the control (-c). The reproducibility of ChIP-seq peaks between replicates was evaluated using the irreproducible discovery rate (IDR) tool (v2.0.2).^88^ The RPKM normalized bigWig files were generated using bamCoverage tool in deepTools (v3.5.1)^83^ with the parameter (– extendReads 120) specified. The input-normalized bigWig files were generated using the bamCompare tool in the deepTools (v3.5.1).^83^ ChIP-seq heatmaps and profiles were generated using the plotHeatmap and plotProfile tools in the deepTools (v3.5.1).^83^ Raw data for publicly available ChIP-seq datasets were downloaded from sources listed in (Table S4) and processed as describe above. Data were visualized using the UCSC Genome Browser.^84^

Coverage plots over the TE consensus sequences were generated using the genomecov function in bedtools (v 2.26.0)^89^ with 1 nt bins and plotted with ggplot2 (v 3.5.1) in R (v 4.4.0). Normalised Tj ChIP coverage across the TE consensus sequences were created using the bamCompare function in deeptools (v 3.5.1) with RPKM normalisation and the log2FC operation. All TE consensus sequences were scaled to 5,000 bits using linear interpolation and the resulting coverage was plotted using ComplexHeatmap (v 2.20.0)^90^ in R (v 4.4.0).

#### Motif discovery and ATAC-seq peak conservation

*De novo* motif analysis of ChIP-seq peaks and known motif enrichments within peak centers were performed using the MEME-ChIP tool (-ccut 100 -dna -order 2 -minw 6 -maxw 15 -streme-pvt 0.05 -streme-align center) with the positional distribution of motifs calculated by CentriMo tool (-centrimo-score 5.0 -centrimo-ethresh 10.0) in MEME Suite (v5.4.1).^91^ Motif scanning was performed using FIMO tool (match *p* < 0.001) in MEME Suite (v5.4.1) (https://meme-suite.org/meme/index.html).^92^ The list of *Drosophila* motifs was downloaded from the FlyFactorSurvey (http://pgfe.umassmed.edu/TFDBS/) database^93^ and lists of known vertebrate motifs for peak central CentriMo enrichments were taken from JASPAR2022_CORE_vertebrates_non-redundant, EUKARYOTE/jolma2013, and MOUSE/uniprobe_mouse motif databases. fastaFromBed (v2.26.0) was used for conversion of bed file coordinates into fasta format using *D. melanogaster* BDGP6.28.dna.toplevel genome. Genomic loci were visualized on the UCSC genome browser.^84^ Orthologous ATAC-seq peak regions between the species genomes were derived using the UCSC LiftOver tool (https://genome.ucsc.edu/cgibin/hgLiftOver).^94^ *D. melanogaster* ATAC-seq peaks were classified as conserved if the peak orthologous LiftOver regions in the *D. yakuba* genome overlapped with the *D. yakuba* ATAC-seq peaks.

#### Quantification And Statistical Analysis

Each experiment of qPCR data relative to *tomato* expression on fresh ovaries was performed 6 times with technical duplicates. Statistical differences were evaluated using Wilcoxon-Mann-Whitney non-parametric test, with corrections for multiple comparisons (Holm–Bonferroni adjustment method) (** indicating a P-value <0.01). Each experiment of qPCR relative to TE expression on ovaries was performed 3 times with technical duplicates. Fold changes of TE from *tj*-SKD compared to *w*-SKD were calculated. Statistical differences between *tj*-SKD and control (*w*-SKD) were evaluated using Wilcoxon-Mann-Whitney non-parametric test using R software. (** indicating a P-value <0.01).

## Supplementary Material

Supplemental information can be found online at https://doi.org/10.1016/j.celrep.2025.115453.

Supplemental Information

## Data Availability

Data: RNA-seq and small RNA-seq data generated in this study have been deposited to GEO database under series GEO: GSE282040. ChIP-seq data reanalyzed in this study have the data accession numbers GEO: GSE97719, ENCSR199TFG, ENCSR414VZP, and GEO: GSE160855. ATAC-seq data reanalyzed in this study have the data accession numbers GEO: GSE233246 and GSE225889. RNA-seq data reanalyzed in this study have the data accession numbers GEO: GSE233246 and GSE225889. The TRAP experiment reanalyzed in this study has the data accession number GEO: GSE230452. Microscopy data reported in this paper will be shared by the lead contact upon request. Code: no original/custom code was generated in this study. Other items: any additional information required to reanalyze the data reported in this paper is available from the lead contact upon request.

## In brief

Alizada et al. report that the transcription factor Traffic jam (Tj) functions as a master regulator of the somatic piRNA pathway in *Drosophila* ovaries. Tj directly controls both piRNA pathway genes and the major piRNA cluster, ensuring transposon silencing in somatic follicle cells and safeguarding the genomic integrity of the oocyte.

## Highlights

- Traffic jam (Tj) acts as a master regulator of the somatic piRNA pathway in *Drosophila*
- Tj regulates piRNA pathway genes, mirroring A-MYB in mouse testis and Ovo in fly ovaries
- *Cis*-regulatory elements with Tj motifs are present at somatic piRNA pathway gene promoters
- The expression of the *flamenco* piRNA cluster is directly controlled by Tj
