# Supplementary material for: The transcription factor Traffic jam orchestrates the somatic piRNA pathway in Drosophila ovaries
Source: Cell Rep. Author manuscript; Available in PMC 2026 Feb 24. (PMC7618790; doi:10.1016/j.celrep.2025.115453)
Supplement: Supplemental Information [file EMS212575-supplement-Supplemental_Information.zip › 1-s2.0-S2211124725002244-mmc1.pdf]

Cell Reports, Volume *44*

Supplemental information

The transcription factor Traffic jam orchestrates the somatic piRNA pathway in *Drosophila* ovaries

Azad Alizada, Aline Martins, Nolwenn Mouniée, Julia V. Rodriguez Suarez, Benjamin Bertin, Nathalie Gueguen, Vincent Mirouse, Anna-Maria Papameletiou, Austin J. Rivera, Nelson C. Lau, Abdou Akkouche, Stéphanie Maupetit-Méhouas, Gregory J. Hannon, Benjamin Czech Nicholson, and Emilie Brasset

Supplementary Figure legends

Figure S1: Knockdown of *tj* in OSCs downregulates somatic piRNA pathway genes (related to Figure 1)

(A) Volcano plot showing differential RNA-seq analysis (DEseq2) between *tj* and *renilla* siRNA knockdowns (96 h; n=3 replicates from distinct samples) in OSCs. Blue dots are showing the soma-enriched piRNA pathway genes (*fs(1)Yb*, *nxf2*, *panx*, *soYb* and *armi*); green dots showing general piRNA factors (e.g., *piwi*); red dots showing *tj*.

(B) Pseudo-palindromic Tj motifs are found within Tj ChIP-seq peaks at promoters of somatic piRNA pathway components and somatic non-piRNA pathway target genes (indicated by asterisk). Motif logos shown above were generated from the shown Tj motif half-sites. Motif logo for MAFA shown below was taken from JASPAR database (MA1521.1).

Figure S1

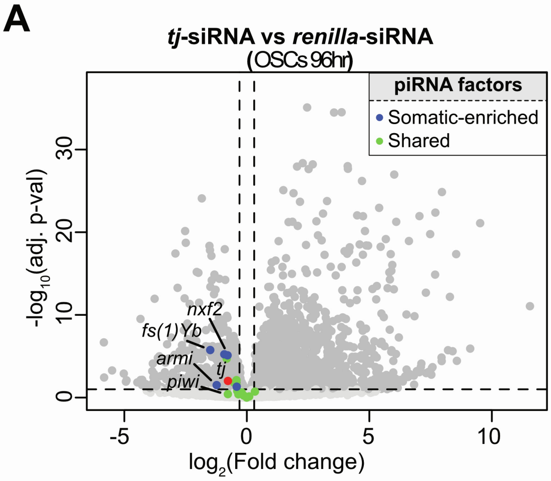
A
tj-siRNA vs renilla-siRNA
(OSCs 96hr)
piRNA factors
Somatic-enriched
Shared
nxf2
fs(1)Yb
armi
tj
piwi
-log10(adj. p-val)
log2(Fold change)
30
20
10
0
-5
0
5
10

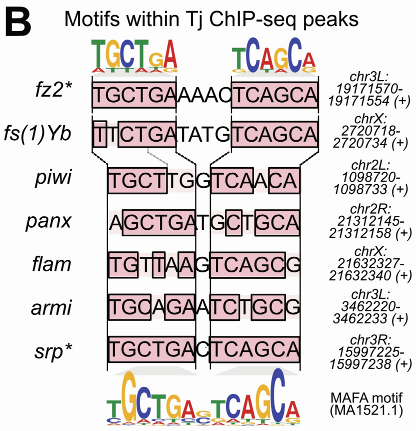
B
Motifs within Tj ChIP-seq peaks
fz2* TGCTGAAAACTCAGCA chr3L: 19171570-19171554 (+)
fs(1)Yb TTCTGATATGTCAGCA chrX: 2720718-2720734 (+)
piwi TGCTTGGTCAACA chr2L: 1098720-1098733 (+)
panx AGCTGATGCTGCA chr2R: 21312145-21312158 (+)
flam TGTTAAGTCAGCG chrX: 21632327-21632340 (+)
armi TGCAGAATCTGCG chr3L: 3462220-3462233 (+)
srp* TGCTGACTCAGCA chr3R: 15997225-15997238 (+)
MAFA motif (MA1521.1)

Figure S2: The +770; +2086 region of *flam* is insufficient for its transcription (related to "Expression of *flam* in follicle cells is dependent on a regulatory region located downstream of its TSS")

(A) Schematic representation of the transgenic constructs.

(B) Confocal images of egg chambers with the indicated genotypes showing Tomato expression (red) by immunostaining. Nuclei are stained with DAPI (white). Scale bars: 10 µm.

Figure S2

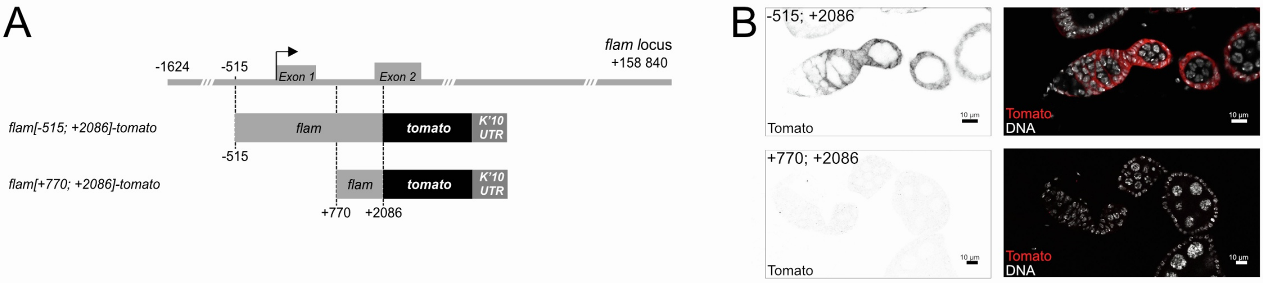
A
flam locus
-1624
-515
Exon 1
Exon 2
+158 840
flam[-515; +2086]-tomato
flam
tomato
K'10 UTR
-515
flam[+770; +2086]-tomato
+770
+2086
B
-515; +2086
Tomato
Tomato
DNA
+770; +2086
Tomato
Tomato
DNA

Figure S3: Tj depletion disrupts *flam* expression (related to Figure 5)

Confocal images of *tj*^{eo2} clonal egg chambers showing *flam* RNA (magenta) and Tj protein (green) levels measured by Immuno-FISH. *tj*^{eo2} clones are circled in white. Nuclei are labelled with DAPI (white). Scale bars: 10 µm.

Figure S3

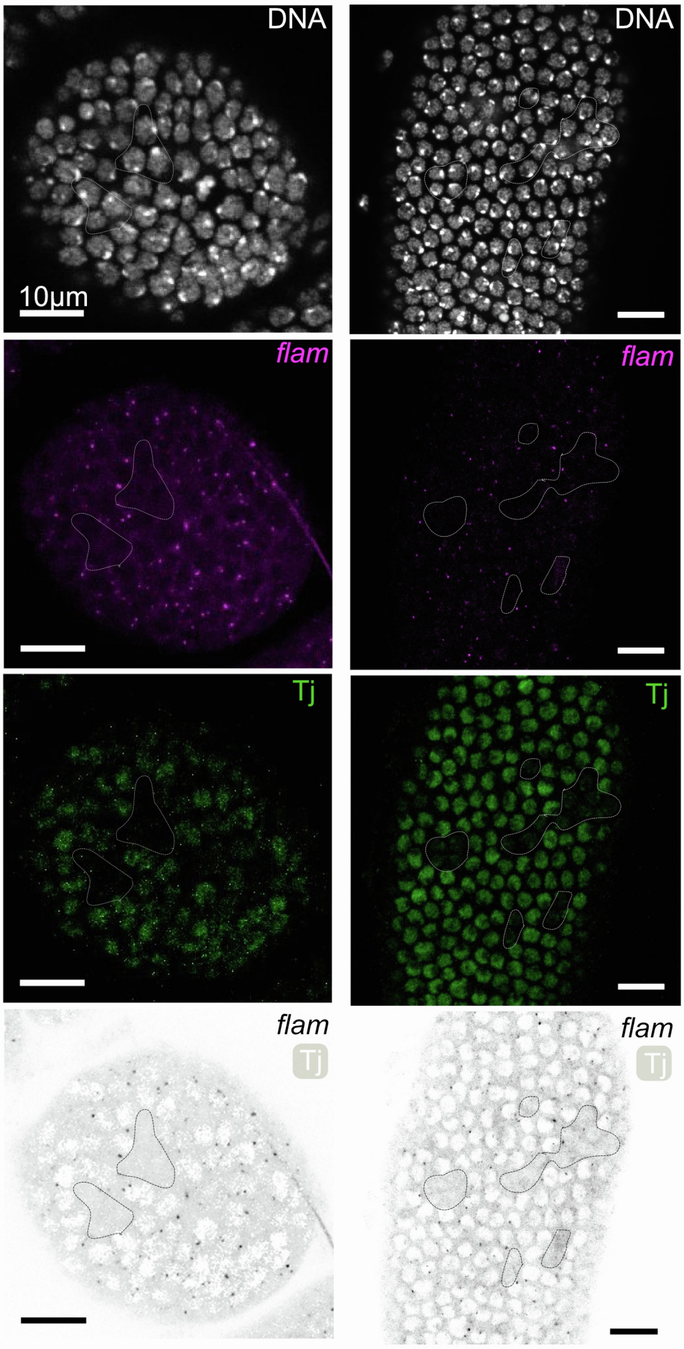

Figure S4: TRAP experiments identify additional candidate TFs regulating *flam* expression (related to “Tj is the master transcription factor for follicle cell-specific expression of *flam”*)

(A) Schematic representation of the Translating ribosome affinity purification (TRAP) method performed on somatic or germ cells of ovaries expressing GFP-tagged ribosomal protein RpL10a, driven by *tj*-Gal4 or *nos*-Gal4 respectively. Comparison between UAS-GFP::Rpl10AGFP; *tj*-Gal4 to UAS-GFP::Rpl10AGFP; *nos*-Gal4 conditions.

(B)-(C). Gene ontology analyses of genes enriched in follicles cells. List of the significantly enriched GO Biological Process **(B)** and Molecular Function **(C)** terms.

Figure S4

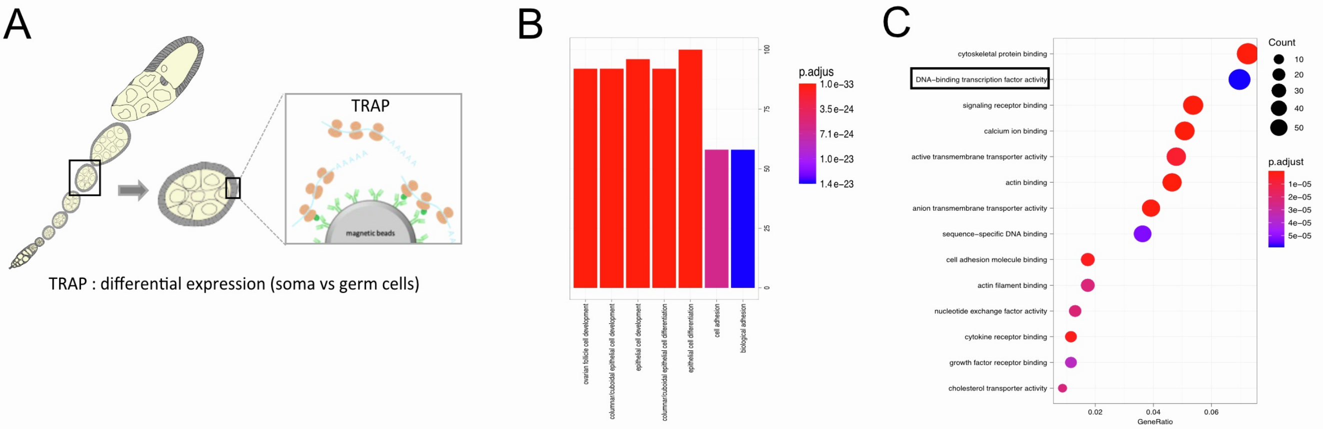
A
TRAP
magnetic beads
TRAP : differential expression (soma vs germ cells)
B
p.adjus
1.0 e−33
3.5 e−24
7.1 e−24
1.0 e−23
1.4 e−23
100
75
50
25
0
ovarian follicle cell development
columnar/cuboidal epithelial cell development
epithelial cell development
columnar/cuboidal epithelial cell differentiation
epithelial cell differentiation
cell adhesion
biological adhesion
C
cytoskeletal protein binding
DNA−binding transcription factor activity
signaling receptor binding
calcium ion binding
active transmembrane transporter activity
actin binding
anion transmembrane transporter activity
sequence−specific DNA binding
cell adhesion molecule binding
actin filament binding
nucleotide exchange factor activity
cytokine receptor binding
growth factor receptor binding
cholesterol transporter activity
0.02
0.04
0.06
GeneRatio
Count
10
20
30
40
50
p.adjust
1e−05
2e−05
3e−05
4e−05
5e−05

Figure S5: Effects of candidates from TRAP experiment on *flam* expression (related to "Tj is the master transcription factor for follicle cell-specific expression of *flam*")

Confocal images of egg chambers with indicated genotype stained for *flam* RNA using smRNA-FISH (magenta). The driver used is *tub-Gal80ts-tj-Gal4*. Nuclei are labelled with DAPI (white). Scale bars: 10 µm.

Figure S5

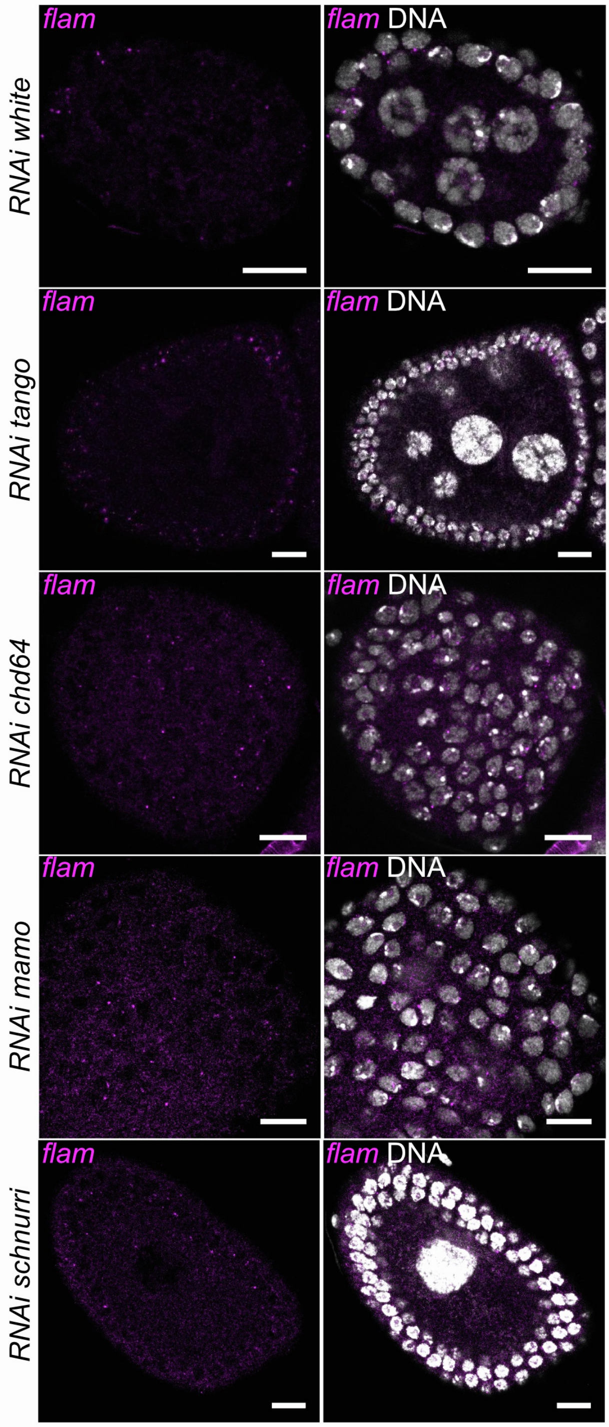

flam
flam DNA
RNAi white
RNAi tango
RNAi chd64
RNAi mamo
RNAi schnurri

Figure S6: **A subset of transposons appears directly regulated by Tj (related to Figure 6)**

(A) Fold-changes in *gypsy* TE expression upon *tj* knockdown in OSCs after 48 h are shown by RT-qPCR (*rpL32* housekeeping control, n=3, error bars indicate standard deviation).

(B) As in **(A)** but comparing fold-changes between 48 h and 96 h of *tj* knockdown

(C) Volcano plot showing upregulation of transposons by 48 h of *tj* siRNA in OSCs using differential mRNA-seq analysis (DEseq2) compared to *renilla* siRNA knockdowns (mRNA-seq, n=3 replicates from distinct samples). Grey circles are showing the genes; blue triangles are showing the transposons.

(D) Fold-changes in TE expression upon *tj*-SKD in ovaries are shown by RT-qPCR (*rpL32* housekeeping control, n=3, error bars indicate standard deviation). Statistical significance was determined using the Wilcoxon-Mann-Whitney test (** indicating a P-value < 0.01).

(E) Confocal images of *tj*-SKD clonal egg chambers showing *mdg1, 412 RNA* (grey), Tj (green) and clonal marker (magenta) by Immuno-FISH. *tj*-SKD clones are circled in white. Nuclei are labelled with DAPI (white). Scale bars: 10 µm.

Figure S6

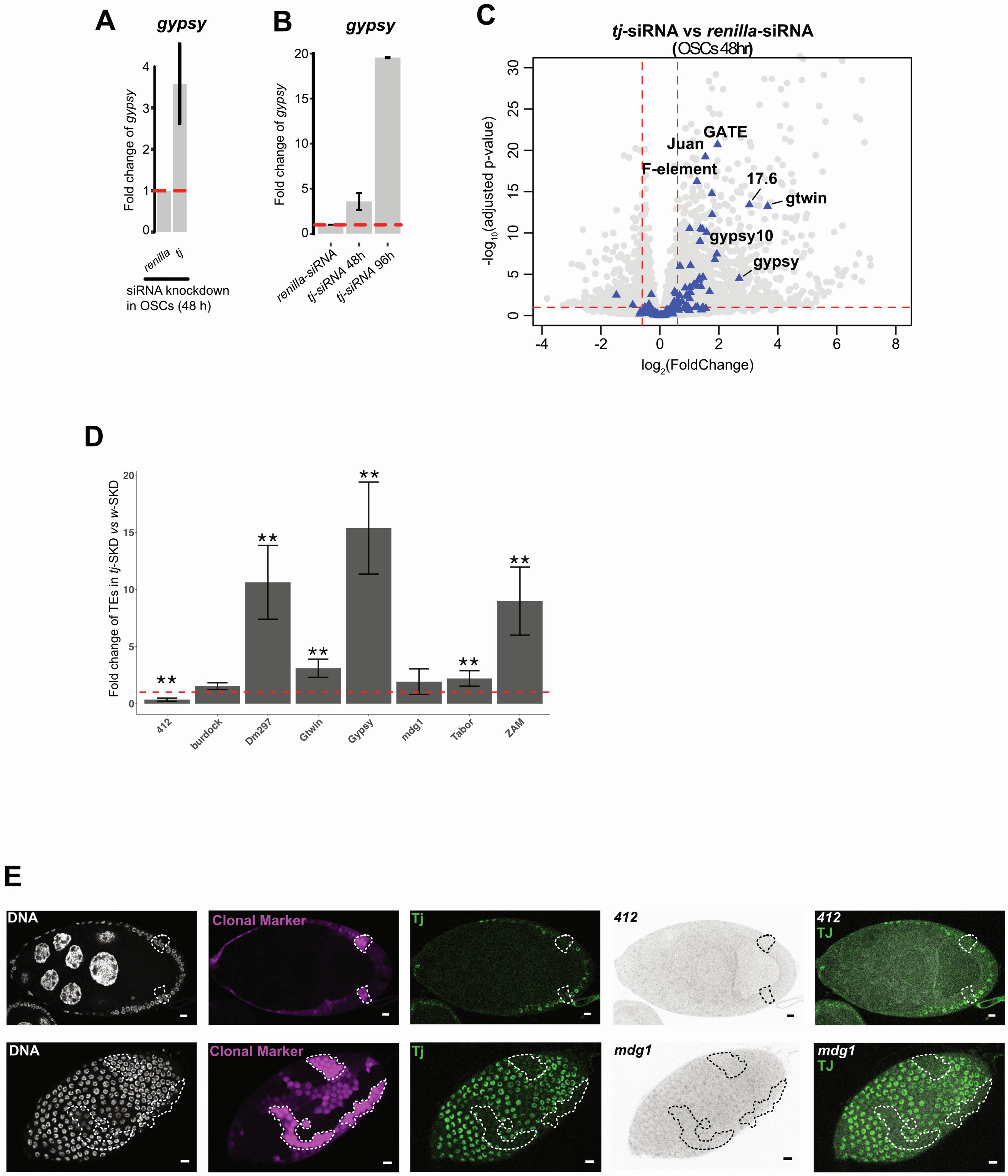

A
gypsy
Fold change of gypsy
renilla
tj
siRNA knockdown in OSCs (48 h)
B
gypsy
Fold change of gypsy
renilla-siRNA
tj-siRNA 48h
tj-siRNA 96h
C
tj-siRNA vs renilla-siRNA (OSCs 48hr)
-log10(adjusted p-value)
log2(FoldChange)
GATE
Juan
F-element
17.6
gtwin
gypsy10
gypsy
D
Fold change of TEs in tj-SKD vs w-SKD
412
burdock
Dm297
Gtwin
Gypsy
mdg1
Tabor
ZAM
E
DNA
Clonal Marker
Tj
412
412 TJ
mdg1
mdg1 TJ

Figure S7: Control of germline and somatic piRNA pathway components relies on Ovo and Tj (related to "Discussion")

(A) Ovo and Tj ChIP-seq showing antagonistic binding at the promoters of germline and somatic genes.

(B) Model depicting the germline and somatic transcriptional regulators of the piRNA pathway components in *Drosophila* ovaries and their putative co-option by transposons for mobilization and infection from soma into the germline.

Figure S7

A

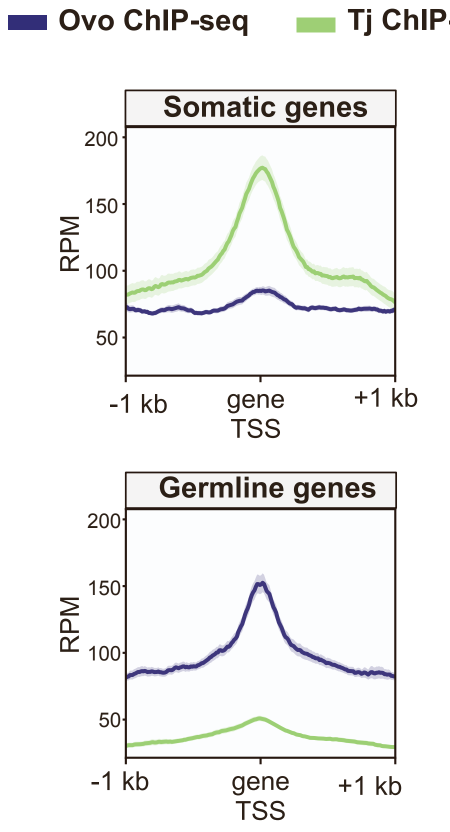
Ovo ChIP-seq
Tj ChIP-seq
Somatic genes
RPM
200
150
100
50
-1 kb
gene TSS
+1 kb
Germline genes
RPM
200
150
100
50
-1 kb
gene TSS
+1 kb

B

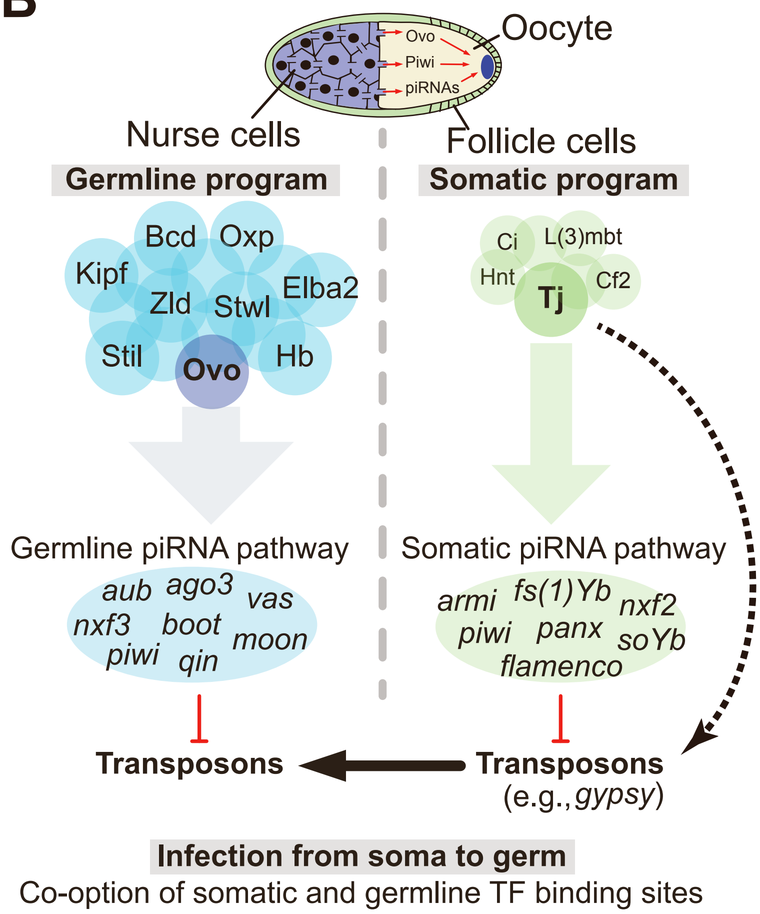
Oocyte
Ovo
Piwi
piRNAs
Nurse cells
Follicle cells
Germline program
Somatic program
Bcd
Oxp
Kipf
Zld
Stwl
Elba2
Stil
Ovo
Hb
Ci
L(3)mbt
Hnt
Tj
Cf2
Germline piRNA pathway
aub ago3 vas
nxf3 boot moon
piwi qin
Somatic piRNA pathway
armi fs(1)Yb nxf2
piwi panx soYb
flamenco
Transposons
Transposons
(e.g., gypsy)
Infection from soma to germ
Co-option of somatic and germline TF binding sites
